# Development of a Vector Set for High or Inducible Gene Expression and Protein Secretion in the Yeast Genus *Blastobotrys*

**DOI:** 10.3390/jof8050418

**Published:** 2022-04-19

**Authors:** Anita Boisramé, Cécile Neuvéglise

**Affiliations:** 1 SPO, INRAE, Institut Agro, Univ Montpellier, 34060 Montpellier, France; cecile.neuveglise@inrae.fr; 2 AgroParisTech, Université Paris-Saclay, 75005 Paris, France

**Keywords:** promoter, xylan, CAzyme, yeast, *Blastobotrys* *yvelinesensis* nomen nudum, cell factory

## Abstract

Converting lignocellulosic biomass into value-added products is one of the challenges in developing a sustainable economy. Attempts to engineer fermenting yeasts to recover plant waste are underway. Although intensive metabolic engineering has been conducted to obtain *Saccharomyces cerevisiae* strains capable of metabolising pentose sugars mainly found in hemicellulose, enzymatic hydrolysis after pretreatment is still required. *Blastobotrys raffinosifermentans*, which naturally assimilates xylose and arabinose and displays numerous glycoside hydrolases, is a good candidate for direct and efficient conversion of renewable biomass. However, a greater diversity of tools for genetic engineering is needed. Here, we report the characterisation of four new promising promoters, a new dominant marker, and two vectors for the secretion of epitope tagged proteins along with a straightforward transformation protocol. The *TDH3* promoter is a constitutive promoter stronger than *TEF1*, and whose activity is maintained at high temperature or in the presence of ethanol. The regulated promoters respond to high temperature for *HSP26*, gluconeogenic sources for *PCK1* or presence of xylose oligomers for *XYL1*. Two expression/secretion vectors were designed based on p*TEF1* and p*TDH3*, two endogenous signal peptides from an α-arabinanase and an α-glucuronidase, and two epitopes. A heterologous α-arabinoxylan hydrolase from *Apiotrichum siamense* was efficiently secreted using these two vectors.

## 1. Introduction

Applications of yeasts in biotechnological processes have a long history, most notably with the use of *Saccharomyces cerevisiae* for the production of fermented beverages or food. Some limitations displayed by *S. cerevisiae* such as poor resistance to osmotic and temperature stress or inefficient protein secretion and modification have resulted in the development of other yeast expression platforms such as *Ogataea polymorpha, Komagataella pastoris*, *Kluyveromyces lactis*, *Yarrowia lipolytica*, and *Blastobotrys adeninivorans* [1,2,3,4]. Strains of genus *Blastobotrys* have not only been found to use a wide spectrum of substances as carbon or nitrogen sources but also to have a strong tolerance to various environmental conditions. Indeed, they display thermo-tolerance, being able to grow at up to 48 °C [5] as well as showing halo- and osmo-tolerance [6]. These overall properties led to the development of commercial applications of the LS3 strain isolated from wood hydrolysates [7]. Recently, on the basis of molecular markers, strain LS3 was reassigned to the species *B. raffinosifermentans* [8], a sibling species of *B. adeninivorans* described in 2007 [9]. LS3 was shown to present three morphological states, with a relationship between temperature and form: under 42 °C, cells harbour a yeast-like form; at 42 °C, they start to form pseudomycelia, before becoming mycelial above 42 °C [5].

After identification and biochemical characterisation of several LS3 genes, genetic tools were developed in the 1990s [10]. The first heterologous gene expression vectors developed for LS3 used *ILV1* and *GAA* promoters [11]. Then, transformation and expression vectors were developed based on the constitutive promoter *TEF1* [12], endogenous auxotrophic markers such as *ILV1* [13] or dominant markers such as the bacterial *hph* gene that confers resistance to hygromycin B [12] and use of 25S rDNA to allow stable integration into nuclear ribosomal DNA. Terentiev et al. designed the first Xplor1 platform based on these elements [14]. Further improvements led to the more versatile Xplor2 system, where yeast selection markers and expression modules are inserted between two 25S rDNA segments, allowing the elimination of bacterial sequences before transformation [15]. Numerous endogenous enzymes of industrial interest have been produced using these platforms, such as the extracellular invertase Inv1 [16], the tannase Tan1 [17] or cutinases Cut1, Cut2 and Cut3 [18]. LS3 was also used for the production of recombinant proteins such as human interferon α2 [19] or *Yarrowia lipolytica* lipase 11 [20]. In parallel, recombinant strains were constructed such as cell bioassays to detect oestrogenic compounds in wastewater at NaCl concentrations as high as 5% [21], or biosensors to detect molecules with progesterone activity [22] or for the rapid diagnosis of a particularly aggressive type of breast cancer [23]. Moreover, LS3 was shown to directly convert starch to ethanol during aerobic and anaerobic cultivation [24] and was subsequently engineered to produce *n*-butanol by fermentation [25]. This capacity deserves to be expanded to include other polysaccharide substrates, such as plant waste.

The recovery of plant biomass, consisting of cellulose, hemicellulose and lignin, is a major global concern. Bacteria and filamentous fungi possess a set of enzymes capable of naturally hydrolysing plant cell walls [26,27]. However, apart from progress made with *S. cerevisiae*, engineering of yeasts to more efficiently degrade these polymers needs further investigation [28]. Xylan, the major hemicellulose in cereals and hardwood, consists of β-1,4-linked D-xylose [29]. This backbone has branched monomers such as L-arabinose and D-glucuronic acid. Fungal enzymes involved in plant polysaccharide degradation are distributed among 35 glycoside hydrolase (GH) families [30]. β-1,4 endoxylanases, involved in the degradation of xylan backbone in smaller oligosaccharides, belong to the GH10 and GH11 families, depending on substrate specificity [31]. Alpha-arabinofuranosidases and arabinoxylan arabino-hydrolases classified in GH43, GH51, GH54 and GH62 families are involved in the release of α-1,2 and α-1,3-linked L-arabinose [32]. Alpha-glucuronidases from the GH67 and GH115 families hydrolyse D-glucuronic acid residues. Acetyl and feruloyl esterases complete the set of enzymes necessary to hydrolyse xylan [33,34]. 

Among Saccharomycotina, *Scheffersomyces stipitis*, a xylose-fermenting yeast isolated in the gut of beetles and termites, was shown to have genes encoding an endoxylanase and an α-glucuronidase of the GH10 and GH115 families, respectively [35]. More recently, *Meyerozyma* and *Trichosporon* species from gut of wood-feeding insects and able to grow on hemicellulosic hydrolysates have been identified [36]. In 2021, Ravn et al. looked for polysaccharide-hydrolysing enzymes in 332 yeast genomes from the Ascomycota phylum and identified several new xylan-degrading species from the Trichomonascaceae family. Notably, they identified surface-anchored xylanases of the GH10 family in several species and reported the presence of a secreted xylanase of the GH11 family in *Blastobotrys mokoenaii* [37]. They highlighted that the eight species from the Trichomonascaceae family have a more diverse and abundant xylanolytic CAZyme distribution than yeasts from other families and classified *B. mokoenaii* as the best xylanolytic yeast.

Based on its promising metabolic capacities, *B. raffinosifermentans* could be used as a cell factory to hydrolyse hemicellulosic polysaccharides and convert released sugars into high value products. The first step to reach this goal is to diversify the availability of relevant genetic tools. In this paper, we describe several promoters and secretory signals in *B. raffinosifermentans* species that could be used for controlled efficient expression and secretion of enzymes of interest. So far, only the promoters p*TEF1* and p*HSB4* have been used as components of expression platforms. The constitutive *TEF1* promoter is a key component of the XploR2 system [15]. The strong constitutive *HSB4* promoter, encoding the histone H4, successfully gave the heterologous expression of both a fluorescent protein and human serum albumin [38]. Here, we characterise five new promoters. One was already described in several yeasts and belongs to the class of strong constitutive promoters, namely the glyceraldehyde-3-phosphate-deshydrogenase p*TDH3* [39]. The four other promoters require specific conditions: stress conditions for the small heat shock protein Hsp26 involved in protein folding and cellular response to heat [40] and the presence of ethanol in *S. cerevisiae*, known to induce the trehalose-6-phosphate synthase 1 promoter (p*TPS1*) [41]. The last two promoters require a specific carbon source such as glycerol for the promoter of the phosphoenolpyruvate carboxykinase 1 (p*PCK1*) and polymers of xylose for the xylosidase 1 promoter (p*XYL1*) [42]. *PCK1* encodes a phophoenolpyruvate carboxykinase involved in the gluconeogenesis pathway and whose expression is repressed by glucose [43]. 

In order to produce heterologous proteins in the external medium, we were interested in developing plasmids with powerful endogenous secretion signals. Several examples of protein secretion are available for *Blastobotrys* species. In the case of heterologous production of *HSA* (human serum albumin) in strain LS3, secretion depends on the native signal sequence of *HSA* [38]. In other constructions, secretion was mediated by the *S. cerevisiae* α-factor secretion signal [3]. In this study, secretion signals belonging to two endogenous glycoside hydrolases were tested. Epitopes to enable identification and purification of recombinant proteins were added in our secretion vectors, either downstream from the signal peptide for N-terminal tagging or upstream from the STOP codon for C-terminal tagging. The two secretion vectors were validated with the heterologous arabinoxylan hydrolase from *Apiotrichum siamense*. Finally, the versatility of the tools was tested in a new *Blastobotrys* species we named *B. yvelinesensis nomen nudum,* which displays a xylanase activity.

## 2. Materials and Methods

### 2.1. Strains and Media

The strains used in this study are listed in Table S1. Genomic DNA extracted from strain LS3 (gifted by Pr. Gotthard Kunze, Leibniz Institute of Plant Genetics and Crop Plant Research, Gatersleben, Germany) was used for the amplification of native promoters and LS3 gene-encoding enzymes. Strain CBS 8335, defined as *B. raffinosifermentans* as LS3 [8], was used as the host for the characterisation of the different promoters and the production of both endogenous and heterologous enzymes. *Apiotrichum siamense* strain L8in5, also isolated from the gut of a beetle in 2016, was used as the donor of DNA for *AXH1* gene amplification. Strain L1-24 of *Blastobotrys yvelinesensis nomen nudum*, isolated in 2018 from the gut of a beetle larvae found in a compost at Les Essarts le Roi in the French region of Yvelines, was used to test the versatility of the developed tools. A second isolate of *B. yvelinesensis,* L2-36, and closely related *Blastobotrys* species were used for taxonomic identification. To this end, the ITS and D1D2 domains of the rRNA gene were amplified and sequenced with primers ITS1, ITS4, NL1, and NL4 (Table S2).

*Blastobotrys* strains were grown in YEA (Yeast Extract 5 g/L, Glucose 15 g/L) supplemented with 75 µg/L of hygromycin or 50 µg/L of nourseothricin for selection of recombinant clones. For growth tests on different sugars, glucose was replaced by 15 g/L xylose or 1.5% glycerol. Medium for L1-24 growth on birch xylan contained 1.7 g/L of N_0_ (Difco, Detroit, MI, USA), 50 mM NH_4_Cl, 50 mM phosphate buffer pH 6.8 and 2% birch xylan from Roth. For recombinant clone selection, 150 µg/L of nourseothricin was added to YEA. For Remazol Brilliant Blue-Xylan (Sigma-Aldrich, Saint-Louis, MI, USA) containing plates, 0.2% of RBB Xylan were mixed with 15 g/L Agar. *A. siamense* L8in5 was grown on YPD medium (Yeast extract 5 g/L, peptone 10 g/L, glucose 10 g/L) for DNA extraction.

### 2.2. Genome Sequencing of A. siamense Strain L8in5 and Identification of GH43 AXH1 Gene

DNA extraction of strain L8in5 was carried out on cells grown in YPD medium to stationary phase, using a previously described in-house protocol involving a mechanical and chemical lysis [44]. A shotgun 400-bp insert library was sequenced using the Illumina HiSeq2000 platform, yielding 6,413,237 pairs of 151-bp reads. Sequencing reads were cleaned with Fastp v0.20.0 with default parameters [45]. *De novo* genome assembly was performed with Spades v3.13.1 with kmer 21,33,55,77,99,127 [46]. An assembly of 23,215,723 bp in 109 scaffolds larger than 5 kb was obtained, with N50 and N90 values of 368,156 bp (L50 = 19) and 132,642 bp (L90 = 59), respectively. The average G + C content was 61.8% for the nuclear genome and 29.0% for the mitochondrion of length 29,548 bp (scaffold88).

To identify a GH43 gene in L8in5, the protein sequence of *Trichosporon asahii* var*. asahii* CBS 2479 XP_014181348 (AXH-like subgroup) was used as a bait for tblastn search on the 109 scaffolds. This strain belongs to the Trichosporonaceae family, as *A. siamense.* Scaffold 54 comprises a gene encoding a 323 amino acid protein with 73% identity and 83% similarity over the entire protein alignment (Scaffold54: complement(6064..7035)).

### 2.3. Accession Numbers

The whole-genome shotgun project of *Apiotrichum siamense* L8in5 was deposited at the NCBI under project PRJNA812413. The Illumina raw reads are available under SRA accession number SRR18212397. The D1D2 and ITS sequence of *B. illinoisensis* YB-1343, *B. malaysiensis* Y-6417, *B. mokoenaii* Y-27120, and *B. yvelinesensis* L1-24 and L2-36 can be found at accession numbers OM904992, OM904990, OM904991, OM904993 and OM904994, respectively.

### 2.4. Promoters Cloning

Six different promoters (p*HSP26*, p*PCK1*, p*TDH3*, p*TEF1*, p*TPS1* and p*XYL1*) were studied (Table 1). The genes were identified in the LS3 genome [47], and we delimited their promoter by fixing the 5’ extremity of the promoter at the extremity of the upstream CDS. The strength and the induction conditions of the promoters were estimated using measurement of eYFP expression. eYFP was amplified using primers 1 and 2 and plasmid JMP1594 as a template [48] and cloned upstream of the *PHO5* terminator of the pBS-SA-p*TEF1*-*PHO5*t plasmid [15] between *Bam*HI and *Not*I (Figure S1). Then, the *TEF1* promoter of pBS-SA-p*TEF1*-eYFP-*PHO5*t was replaced at the *Sal*I and *Bam*HI restriction sites by the 5 other promoters amplified from LS3 genomic DNA using primers 3 to 12. The six expression cassettes were inserted at the *Sal*I and *Apa*I sites in the plasmid pARE12 containing the *hph* gene that confers hygromycin B resistance [49]. The expression cassette and the selection marker are flanked by 25S rDNA regions to allow stable integration into the ribosomal DNA region [12,15]. All plasmids are listed in Table S3 and the primers in Table S2. The *Asc*I restriction fragments were then inserted into the CBS8335 strain using a LiAc method detailed below. Additionally, a 1.7 kb *Aat*II/*SalI*I restriction fragment containing the *hph* gene under the control of the hp4d promoter [50] was subcloned at the *Aat*II/*Sal*I site of the pBS-SA-p*TDH3*-eYFP-*PHO5*t plasmid and the resulting vector was linearized at the unique *Bsu*36I site in p*TDH3* to allow integration of the expression cassette at the *TDH3* locus (Figure S1). Correct integration in hygromycin resistant clones was checked using primer 13, which was hybridized upstream of the *TDH3* cloned region, and primer 14, which recognized part of the *PHO5* terminator.

### 2.5. Construction of Epitope Tagging and Secretion Plasmids

Two endogenous glycosyl hydrolases were used as templates for the construction of secretion vectors. First, the ARAD1D18216g coding sequence that matches α-arabinanases (*ABN1* of the GH43 family, File S1) was expressed under the control of the *TDH3* promoter. Four primers were designed (16 to 19) to N-terminally tag the mature form of this enzyme and allow further cloning of enzymes of interest downstream to the N-terminal signal sequence of the arabinanase to target them to the secretory pathway. A 52 nt long sequence, encoding the V5 epitope (GKPIPNPLLGLDST), was inserted between amino-acid 22 and 23, followed by a *Spe*I restriction site. The SignalP prediction site indeed identified the site of cleavage by the signal peptide protease between the two alanines in position 22 and 23 [51]. NetNGlyc tool was used to predict N-glycosylation sites [52]. Primers 17 and 18 that correspond to the V5 epitope in their 5’ extremities overlapped over 18 nucleotides. The 0.1 kb long amplified fragment using primers 16 and 17 and the 1.1 kb long amplified fragment using the primers 18 and 19 were fused and further amplified using the two external primers (16 and 19). The complete fragment was cloned in the pBS-SA-p*TDH3*-eYFP-*PHO5*t at the *Bam*HI and *Not*I sites in place of the eYFP (Figure S2A). The nourseothricin acetyl transferase (nat) resistance cassette with the *Y. lipolytica TEF1* promoter and *LIP2* terminator (Tristan Rossignol, personal gift) was inserted as a *Nsi*I fragment in the unique *Pst*I site of the transitional vector. The unique *Nsi*I site in the *TDH3* promoter was used to linearize the final plasmid pBS-SA-p*TDH3-V5-*Br*ABN1*-*PHO5*t + nat before transformation. Correct integration in nourseothricin resistant clones was checked using primer 13, hybridizing upstream of the *TDH3* cloned region, and primer 14, which recognized part of the *PHO5* terminator.

The second CDS, ARAD1D23848g that putatively encodes an α-glucuronidase (*AGU1* of the GH67 family, File S2), was expressed under the control of the *TEF1* promoter. A 6xHis tag was added downstream of the alanine at position 24 that corresponds to the predicted site of cleavage by the signal peptidase. Primers 20 and 21 and primers 22 and 23 allowed the amplification of the first 24 codons and of the last 831 codons, respectively. Since both primers 21 and 22 contained a 6xHis epitope sequence surrounded by a *Bss*HII and a *Pml*I restriction sites, the two fragments were hybridized and the entire CDS was amplified with primers 20 and 23 before cloning at the *Bam*HI/*Not*I sites of the pBS-SA-p*TEF1*-eYFP-*PHO5t* vector (Figure S2B). The *hph* resistance cassette was further inserted in this plasmid between *Nae*I and *Sal*I. The unique *Hpa*I site in the *AGU1* CDS was used to linearize the pBS-SA-p*TEF1*-6xHis-Br*AGU1*-*PHO5*t + hph plasmid and target insertion at the *AGU1* locus. Correct integration in hygromycin resistant clones was checked using primer 24, hybridizing downstream of the *AGU1* coding sequence, and primer 25, which recognized the *AGU1* signal sequence.

All genetic fusions were validated by DNA sequencing.

### 2.6. Heterologous Enzyme Cloning and Expression

A GH43 belonging to the AXH-like subgroup was identified in *A. siamense*. It perfectly aligned with other AXH-like proteins (File S3). Two primers, 26 and 27, were designed to amplify and clone the *A. siamense* gene in both the pBS-SA-p*TDH3*-V5-*ABN1*-*PHO5*t plasmid downstream of the Abn1 signal peptide coding sequence and the V5 epitope, and the pBS-SA-p*TEF1*-SS-6xHis-*AGU1*-*PHO5*t plasmid downstream of the *AGU1* signal peptide coding sequence (Figure S3). The amplified fragment was inserted either at the *Spe*I/*Not*I sites of the first plasmid or at the *Bss*HII/*Not*I site of the second plasmid. A 24 nt long sequence encoding the FLAG tag (DYKDDDDK) was included in primer 27 to express a C-terminally tagged enzyme. To favour integration of the latter plasmid, a 1.1 kb long DID2 + ITS region of the LS3 rDNA amplified with ITS1 and NL4 primers was cloned at a *Eco*RV site of the pBS-SA-p*TEF1*-SS-*AXH1*-Flag-*PHO5*t plasmid and the resulting vector was linearised by *Bst*BI to target integration at the rDNA loci.

### 2.7. Protocol for Blastobotrys *spp.* Transformation

A loop of yeast cells freshly grown on solid YEA overnight at 30 °C was suspended in 1 mL of 0.1 mM LiAc pH 8.5 and incubated for one hour at 30 °C. Competent cells were concentrated five-fold by centrifugation 5 min at 4000 rpm, suspended in the same solution and stored at 4 °C from 2 h to 72 h. For transformation, 100 µL of competent cells was added to 10 µL of Carrier DNA (Clontech, Takara Bio, San Jose, CA, USA) and 200 µg of transformant DNA. An amount of 700 µL of 40% PEG in 0.1 mM LiAc pH 8.5 was then added and the suspension mixed before incubation at 30 °C for one hour. The transformation mixture was then heat shocked for 20 min at 42 °C before a 4 min centrifugation at 4000 rpm. The pelleted cells were suspended in 400 µL of H_2_O and plated on selective solid media. 

### 2.8. Flow Cytometry

For flow cytometry analysis, cells grown in YEA medium containing different sugars at 30 °C under 180 rpm agitation were suspended in phosphate buffer saline buffer (PBS) at 10^6^ cells per mL. YFP fluorescence was determined using a C6 Accuri (Ann Arbor, MI, USA) flow cytometer with an excitation wavelength of 488 nm and a 533/30 nm emission filter. Acquisition was performed on 20,000 events observed with a gating on forward scatter/side scatter signal. The flow rate was set to approximately 2000 events per second (medium flow, 35 μL/min; core, 16 μm).

### 2.9. Microscopy

Cells were examined by fluorescence microscopy (Olympus BX51) with 460 to 490 nm excitation and 520 nm emission filters using an Olympus 100× oil immersion objective and 10× oculars.

### 2.10. Protein Analysis

For intracellular protein extracts, cell pellets were washed in PBS and suspended in PBS containing protease inhibitors (complete EDTA-free from Roche) and disrupted mechanically with glass beads in a Bead-Beater 24™ (MP Biomedicals, Irvine, CA, USA) in four rounds of 20 s each with 5 min incubation in ice between each round. The lysates were collected following centrifugation at 17,000× *g* for 10 min at 4 °C. For secreted proteins, culture supernatants were harvested by centrifugation and concentrated using Amicon ^®^ Ultra—0.5 mL units 30 K or 50 K (Merck Millipore, Burlington, MA, USA). For the V5-tagged Abn1p, an endoH treatment was performed (New England Biolabs, Ipswich, MA, USA). Following separation on NuPAGE 10% or 4–12% (Invitrogen, Carlsbad, CA, USA), proteins were either transferred onto a nitrocellulose membrane (Amersham Protran, Cytiva, Chicago, IL, USA) for Western blotting or stained with EZ-blue (Sigma-Aldrich, Saint-Louis, MI, USA). For blotting, membranes were rinsed in PBS and blocked in PBST (PBS + 0.1% Tween 20 + 2% skim milk from Difco) for 1 h at room temperature. The membranes were then incubated overnight at 4 °C in PBST containing a 1:5000 dilution of either a monoclonal anti-FP antibody (Clontech, Takara Bio, San Jose, CA, USA), a monoclonal anti-V5 antibody (Invitrogen, Carlsbad, CA, USA) or a monoclonal anti-Flag antibody (Sigma-Aldrich, Saint-Louis, MI, USA). After three washes in PBST, a 1 h incubation in the presence of either peroxidase-conjugated anti-mouse IgG antibodies (GE Healthcare, Chicago, IL, USA) was performed. The membranes were washed three times before detection of the signal using the Enhanced chemiluminescence ECL Plus^TM^ detection system (GE Healthcare, Chicago, IL, USA).

## 3. Results

### 3.1. B. raffinosifermentans Is Partially Equipped to Degrade Xylan

To decipher if *B. raffinosifermentans* has the potential to degrade hemicelluloses such as xylan and liberate free sugars such as arabinose or xylose, we used the data of the MycoCosm fungal genomics portal, which annotated 87 coding sequences as glycosyl hydrolases in the LS3 genome [53]. We focused on GH3, GH10/11, GH43 and GH67 families that gather enzymes capable of debranching arabino- or glucurono-xylan (GH43 and GH67 families, respectively) and degrading the xylan backbone β-1,4 endo-xylanases (GH10/11) or β-1,4-xylosidases (GH3). As listed in Table 2, the ARAD1D18216g sequence encodes an enzyme belonging to the GH43 family. The protein displays higher similarity with Abn1 proteins (File S1) that have α-1,5 endo-arabinanase activity and may thus more likely degrade the arabinan backbone. 

The second interesting enzyme, encoded by ARAD1D23848g, corresponds to an α-glucuronidase of the GH67 family (File S2) whose activity consists in removing α-1,2-linked 4-O-methyl glucuronic acid from xylans. 

The last glycosyl hydrolase activity that may be encoded in the genome of LS3 is a β-xylosidase GH3 (File S4) with two different CDS, ARAD1D50644g and ARAD1C05676g, suggesting that this yeast has the capacity to release a xylose unit from the non-reducing end of the xylan backbone or xylose oligomers. The four glycosyl hydrolases are N-glycosylated secreted proteins with up to ten predicted positions in Agu1. Two feruloyl esterases were also detected in the LS3 genome: ARAD1A06094g (Tan1) and ARAD1A19822g (File S5) [17,54]. 

Neither a β-1,4 endo-xylanase (GH10, GH11) nor a α-1,2 or α-1,3 arabinofuranosidase (such as GH43 AXH) was found in the genome of LS3. Among the Trichomonascaceae family, two other species, *Trichomonascus ciferrii* and *Sugiyamaella lignohabitans,* have all or part of this enzymatic set in their genome (Table 2). No member of the GH43 AXH family was detected in these two species, but in contrast to LS3, sequences corresponding to endo-xylanases of the GH10 family were found.

### 3.2. Growth Capacities of B. raffinosifermentans CBS 8335

**Figure 1:**
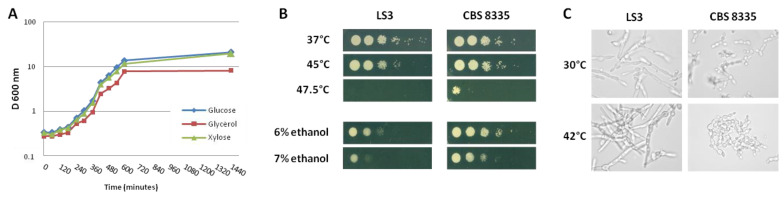
CBS 8335 carbon source use and tolerance to stress. (**A**) Growth curves of CBS 8335 grown in glucose (diamond), xylose (triangle) and glycerol (square). (**B**) Resistance to high temperature and ethanol stresses. Serial 1:20 dilutions (5 μL) of *B. raffinosifermentans* LS3 and CBS 8335 strains were spotted on YEA plates or YEA plates containing 6% or 7% ethanol. Plates were incubated at indicated temperatures (for heat stress) or at 30 °C (for ethanol stress) for 24 h. (**C**) Cell visualisation. Overnight grown LS3 and CBS 8335 cells at 30 °C were either directly observed or incubated for 8 h at 42 °C before observation using an Olympus BX51 microscope.

As the *B. raffinosifermentans* CBS 8335 strain was used experimentally in this study, we first confirmed that this strain had the capacity to grow on carbohydrates of interest, i.e., glucose, glycerol and xylose (Figure 1A). To identify the metabolic pathway used by *B. raffinosifermentans* to assimilate xylose, blastP was performed using the *S. stipitis* coding sequences for xylose-reductase XR (PICST_89614), xylitol dehydrogenase XDH (PICST_86924) and xylulose kinase XK (PICST_68734) as queries to identify LS3 homologues. Results presented in Table 3 clearly indicate that LS3 contains in its genome the set of enzymes required to assimilate xylose through the oxidative-reductive pathway, with the presence of both XR and XDH enzymes, which convert xylose to xylulose subsequently phosphorylated by XK to enter the pentose phosphate pathway.

We also tested the tolerance of CBS 8335 to different stresses. As shown in Figure 1B, CBS 8335 grew at a temperature up to 47.5 °C, whereas growth of LS3 was inhibited at this temperature. CBS 8335 was also more tolerant to ethanol than LS3, being able to grow even in a 7% ethanol containing medium. In contrast, LS3 formed mycelium at elevated temperature (Figure 1C), whereas CBS 8335 still showed a yeast morphology at 42 °C in rich medium.

### 3.3. Characterisation of the New Promoters on YEA Medium at 30 °C

In order to enable degradation of xylan by *Blastobotrys* species, the idea was to replace the promoters of native genes with constitutive strong promoters and to complete the endogenous enzymatic set with exogenous genes. Thus, new promoters to control gene expression had to be developed (Table 1). To this end, we analysed five new promoters from LS3 and compared them to the *TEF1* promoter used in the Xplor2 system [15]. For each of these, we defined their strength and conditions for expression using a yellow fluorescent protein (YFP) as a reporter.

**Figure 2:**
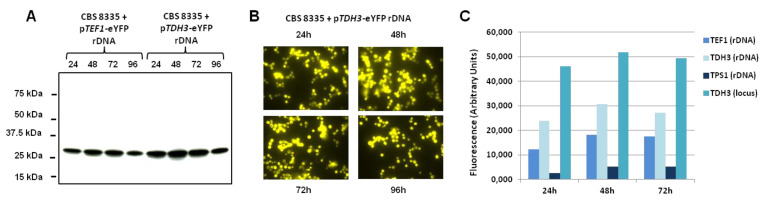
Time-course expression of YFP under the control of *TEF1*, *TDH3* or *TPS1* promoters. (**A**) Two selected CBS 8335 recombinant clones expressing the fluorescent protein under the control of the *TEF1* promoter (left) or the *TDH3* promoter (right) were grown at 30 °C in YEA and 1 mL aliquots were collected at 24, 48, 72 and 96 h. Intracellular protein extracts were analysed by SDS-PAGE and Western blotting using the monoclonal anti-FP antibody (Clontech). The numbers on the left indicate the molecular masses of the protein standards (Precision Plus Protein™ Standards from Bio-Rad). (**B**) YFP fluorescence visualisation. Cells expressing the YFP protein under the control of the *TDH3* promoter were observed after 24, 48, 72 and 96 h of culture using an Olympus BX51 microscope as described in Material and Methods. (**C**) Flow cytometry analysis of YFP expression for CBS 8335 recombinant clones containing the p*TEF1-YFP*, the p*TDH3-YFP* and the p*TPS1-YFP* cassettes integrated either in the rDNA or at the *TDH3* locus for the p*TDH3-YFP* construction. Aliquots of cultures grown for 24, 48 and 72 h in YEA at 30 °C were resuspended in PBS at 10^6^ cells per mL. YFP fluorescence was determined using a C6 Accuri cytometer. Results correspond to one representative experiment of four.

In a first attempt, we characterised the *THD3* promoter and compared it to the *TEF1* promoter. A time course experiment from 24 to 96 h of culture at 30 °C in yeast extract medium containing 15 g/L of glucose was performed for two recombinant strains of CBS 8335. As shown in Figure 2A, levels of the fluorescent protein expressed were higher when eYFP expression was under the control of the *TDH3* promoter than with the p*TEF1* promoter for similar amounts of total protein in cell lysate (Figure S4). For both promoters, maximal amounts of YFP were obtained after 48 h of culture, and maintained for at least a further 24 h, followed by a slight decrease detected at 96 h (Figure 2A). The behaviour of the *B. raffinosifermentans TDH3* promoter as a strong constitutive promoter was confirmed by fluorescence microscopy images of yeast cells after 24 h to 96 h of culture (Figure 2B).

To obtain more precise data, YFP levels measured by flow cytometry confirmed the kinetics of expression with a maximum of fluorescence detected after 48 h of culture (Figure 2C). The fluorescence intensity was 2 to 1.5 higher under the control of the *TDH3* promoter than with the *TEF1* promoter at 24 h and 72 h, respectively. Flow cytometry analysis in the same growth conditions showed that among the other four promoters tested, only the *TPS1* promoter gave detectable YFP fluorescence, with a maximum after 48 h of culture. However, the maximal level was only 30% of that obtained with p*TEF1* (Figure 2C). No activity was detected for *HSP26*, *PCK1* and *XYL1* on YEA medium at 30 °C.

Since the *TDH3* promoter region is long enough (1153 pb) to target integration through homologous recombination, we tested integration at the *TDH3* locus through linearization of the plasmid in the middle of the *TDH3* promoter. Correct integration at the *TDH3* locus was observed in about 20% of the transformants (2 clones out of 11). A similar time course showed that YFP was expressed at higher levels than after integration in the rDNA. Indeed, fluorescence intensity was up to two-fold higher after 24 h when the p*TDH3*-eYFP cassette was targeted to the *TDH3* locus compared with rDNA integration, and 3.8-fold higher compared with the rDNA-integrated p*TEF1*-eYFP cassette (Figure 2C).

### 3.4. Characterisation of Inducible Promoters

**Figure 3:**
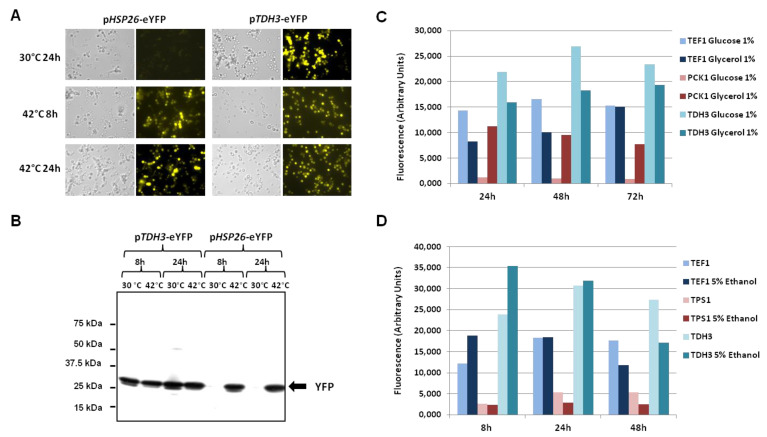
YFP expression in stress conditions under the control of *HSP26, PCK1, TEF1, TDH3* or *TPS1* promoters. (**A**) CBS 8335 recombinant clones expressing the fluorescent protein under the control of the *HSP26* promoter (left) or the *TDH3* promoter (right) were grown in YEA for 16 h at 30 °C. Half of each culture was incubated at 42 °C. Fluorescence was visualised using an Olympus BX51 microscope after 8 h and 24 h of incubation and compared to the control culture at 30 °C for 24 h. (**B**) Anti-FP Western blotting of intracellular protein extracts from cell samples withdrawn for each condition: 30 °C and 42 °C; 8 h and 24 h. The numbers on the left indicate the molecular masses of the protein standards (Precision Plus Protein™ Standards from Bio-Rad). (**C**) Flow cytometry analysis of YFP expression for CBS 8335 recombinant clones containing the p*TEF1*-YFP, the p*PCK1*-YFP and the p*TDH3*-YFP cassettes integrated in the rDNA, grown either in YEA containing 1% glucose or 1% glycerol instead of glucose at 30 °C during 24, 48 and 72 h. YFP fluorescence was determined using a C6 Accuri cytometer. Results correspond to one representative experiment of three. (**D**) Flow cytometry analysis of YFP expression for CBS 8335 recombinant clones containing the p*TEF1*-YFP, the p*TPS1*-YFP and the p*TDH3*-YFP cassettes integrated in the rDNA. After 16 h incubation in YEA, 5% ethanol was added in half of each culture. Incubation was extended for 8, 24 and 48 h. YFP fluorescence was determined using a C6 Accuri cytometer. Results correspond to one representative experiment of three.

To define the optimal conditions for induction of the *HSP26* promoter, an rDNA integrated CBS 8335 clone was cultivated at 42 °C for 8 and 24 h and monitored by fluorescence microscopy. As shown in Figure 3A, while no fluorescence was detected after 24 h at 30 °C when YFP expression depended on the p*HSP26* promoter, some fluorescence was detected after 8 h at 42 °C and maintained for at least a further 16 h. The intensity of fluorescence was similar to that observed for p*TDH3*-controlled YFP expression at 30 °C. For this construct, the level of YFP slightly decreased at 42 °C. Western blot analysis of intracellular extracts gave results in accord with fluorescence microscopy (Figure 3B). These results show that expression due to p*HSP26* is inducible at high temperature.

Since *B. raffinosifermentans* can grow on glycerol as the sole carbon source [8], and glycerol is a substrate for mitochondrial oxidation and gluconeogenesis, we evaluated YFP expression in a recombinant CBS 8335 clone that contained the p*PCK1*-eYFP cassette integrated into its rDNA, cultivated in 1% glucose or in 1% glycerol. As shown in Figure 3C, a basal level of fluorescence was detected during the 72 h of incubation in the presence of glucose. In contrast, a 10-fold induction of YFP expression was observed after 24 h of growth in the presence of glycerol. The fluorescence intensity was slightly higher than that measured with the *TEF1* promoter in the same medium, but less than that obtained with p*TDH3*. After 48 and 72 h of culture in glycerol, the p*PCK1*-controlled YFP expression tended to decrease while levels of fluorescence increased for the p*TEF1* and p*TDH3*-eYFP constructs, reaching their maximum after 72 h of growth. Although slightly lower than the values measured in glucose, p*TDH3*-dependent YFP expression was the strongest in glycerol at all times.

In order to evaluate the activity of these different promoters after fermentation, we measured YFP expression in a 5% ethanol containing medium (Figure 3D). Levels of YFP were higher for both the p*TEF1* and p*TDH3* reporter constructions after 8 h of culture in the presence of ethanol in comparison with the basal YEA medium and no change was observed for the *TPS1* promoter. After 24 h, YFP expression was similar in both media for the two strong constitutive promoters and slightly lower after 48 h in 5% ethanol. After 24 and 48 h, p*TPS1*-controlled YFP expression was reduced by about 50%, indicating that the *TPS1* promoter is repressed due to osmotic stress induced by ethanol. This regulation is different from that in *S. cerevisiae* [41].

### 3.5. Expression of Endogenous Epitope-Tagged Secretory Proteins

With the aim of developing vectors for the secretion of heterologous proteins, we assessed the efficiency of signal sequences from endogenous secreted proteins. For this purpose, we selected the two secreted glycosyl hydrolases putatively involved in the degradation of pectin or hemicellulose through their debranching activity: namely, Abn1 (ARAD1D18216g) of the GH43 α-arabinanase-like family and Agu1 (ARAD1D23848g) of the GH67 α-glucuronidase family (Table 2). In order to detect and/or purify the secreted proteins, epitopes were added at the N-terminal of the mature proteins, i.e., a V5-tag and a 6xHis-tag, respectively. Expression of Abn1 and Agu1 were controlled by the *TDH3* and *TEF1* promoters, respectively (Table S3).

**Figure 4:**
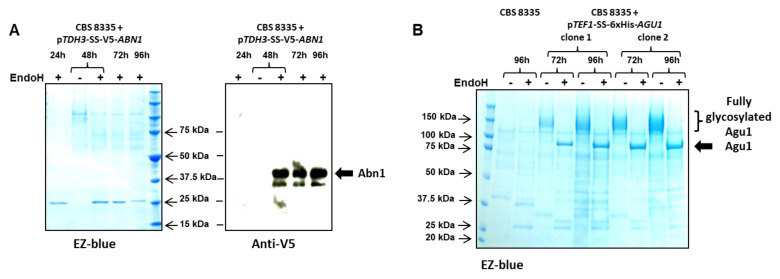
Endogenous protein secretion using the two constitutive p*TDH3* and p*TEF1* promoters. (**A**) Time course of expression and secretion of the V5-tagged Abn1 protein under the control of the p*TDH3* promoter. A *TDH3*-targeted CBS 8335 recombinant clone was grown at 42 °C in YEA and 1 mL samples of supernatant collected at 24, 48, 72 and 96 h and concentrated before endoglycosidase H treatment. After SDS-PAGE, total proteins were detected using EZ-blue reagent (left panel) and Abn1p revealed through an anti-V5 blot (right panel). (**B**) Secretion of the Agu1 protein under the control of the p*TEF1* promoter. Two *AGU1* targeted CBS 8335 recombinant clones were grown at 42 °C in YEA and 1 mL samples of supernatant were collected at 72 and 96 h, concentrated before endoglycosidase H treatment. After SDS-PAGE, total proteins were detected using EZ-blue reagent from SIGMA. Precision Plus Protein™ Standards (Bio-Rad) were used for gel calibration.

In a first attempt, a CBS 8335 recombinant clone carrying the p*TDH3*-SS-V5-*ABN1*- expression cassette at the *TDH3* locus was cultivated for 72 h at 30 °C. An anti-V5 blot performed on concentrated and deglycosylated supernatants gave very weak signals We repeated the experiment at 42 °C. Samples were withdrawn after 24, 48, 72 and 96 h of incubation and concentrated supernatants were analysed by Western blotting. As illustrated in Figure 4A, no signal was observed after 24 h. An incubation of 48 h was required to detect Abn1 in the supernatant and the signal remained stable for an additional 48 h. Detection of the V5-tagged Abn1 was possible only when the protein had undergone a deglycosylation step through endoglycosidase H treatment. This observation confirmed that Abn1 was glycosylated as predicted by the NetNGly server, i.e., with 4 potential N-glycosylation sites at positions N_49_, N_61_, N_135_ and N_201_ (File S1). Furthermore, since two bands were observed after endo-H hydrolysis, we hypothesise that the N-linked oligosaccharides underwent complex processing in the Golgi apparatus of CBS 8335.

The second enzyme, Agu1, was expressed at 42 °C under the control of the *TEF1* promoter integrated at the *AGU1* locus. A strong signal at approximately 150 kDa was observed in the growth medium for two recombinant clones after 72 h and 96 h of growth and was not detected in the supernatant of CBS 8335 (Figure 4B). The apparent molecular mass of the detected protein was 85 kDa when samples were treated with endoglycosidase H, as expected for the deglycosylated recombinant Agu1 protein. Attempts to detect the 6xHis-tagged Agu1 recombinant protein using N-terminal His-tag antibodies failed even after EndoH treatment. This may be due to the presence of an arginine upstream of the His-tag following the peptide signal cleavage or to the presence of residual sugars on the 10 predicted N-glycosylation sites that mask the His-tag (File S2).

### 3.6. Expression of a Heterologous Glycosyl Hydrolase of the GH43 AXH-like Subgroup

**Figure 5:**
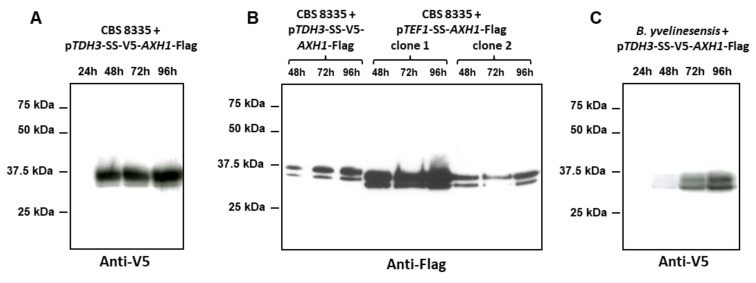
Expression and secretion of heterologous proteins using the two vectors and expression in a new *Blastobotrys* species. (**A**) Time course of expression and secretion of the V5-tagged Axh1 protein under the control of the p*TDH3* promoter. A *TDH3* targeted recombinant clone was grown at 42 °C in YEA and 1 mL aliquots of supernatant were collected at 24, 48, 72 and 96 h and concentrated before SDS-PAGE. Axh1p was revealed using an anti-V5 blot. (**B**) Secretion of the Axh1 protein under the control of the p*TEF1* promoter. Two rDNA targeted recombinant clones were grown at 42 °C in YEA and 1 mL aliquots of supernatant were collected at 48, 72 and 96 h and concentrated before SDS-PAGE. Axh1p was revealed using an anti-Flag blot and compared with the p*TDH3*-controlled Axh1 expression. (**C**) Time course of expression and secretion of the V5-tagged Axh1 protein under the control of the p*TDH3* promoter in a *B. yvelinesensis* isolate. The recombinant clone was grown at 30 °C in YEA and 1 mL aliquots of supernatant were collected at 24, 48, 72 and 96 h and concentrated before SDS-PAGE. Axh1p was revealed using an anti-V5 blot.

To confirm the efficiency of the two selected signal sequences for heterologous protein secretion, we constructed two expression plasmids designed to express a GH43 AXH-like enzyme. The coding sequence was amplified from an *A. siamense* isolate L8in5 obtained from the gut of a cetoniae larvae. The genome was sequenced and the assembly was searched for homologues of *T. asahii var. asahii* GH43 AXH-like protein. The predicted AsAxh1 protein of L8in5 is highly conserved compared with arabinoxylan arabinofuranosidases from various Basidiomycetes and Ascomycetes (File S3). As for Abn1 proteins, the GH43 AXH-like enzymes displayed the three catalytically active acidic residues required for their activity [55]. Interestingly, all Axh1p collected in databases are devoid of a conventional signal peptide. As a consequence, no N-glycosylation site was predicted. Here, we intended to use this protein to validate our expression secretion vectors. Therefore, two expression cassettes were constructed: in the first one, the C-terminally Flag-tagged AsAxh1 was fused to the Abn1 signal sequence and the V5 tag and for the second one, the C-terminally Flag-tagged AsAxh1 was cloned downstream of the *TEF1* promoter and the Agu1 signal sequence. The first plasmid was integrated at the *TDH3* locus of CBS 8335, and the second one was targeted in the CBS 8335 rDNA (Figure S3). Recombinant clones for each of the expression constructions were grown for 96 h at 42 °C in YEA. As shown in Figure 5A, the heterologous V5-tagged Axh1 protein was efficiently produced and secreted in the medium after 48 h of culture and was accumulated during the following 48 h. The expression and secretion kinetics were thus similar to those described for the p*TDH3*-controlled endogenous Abn1 protein.

An anti-Flag blotting was performed for samples withdrawn after 48, 72 and 96 h for the two recombinant clones transformed with the p*TEF1-SS-AXH1*-Flag construction and compared to the p*TDH3*-SS-V5-*AXH1*-Flag expressing clone (Figure 5B). Interestingly one of the two clones secreted larger amount of Axh1 at all times (from 48 h to 96 h), while levels of Axh1 obtained for the second clone were comparable to that observed for p*TDH3-*controlled *AXH1* expression. This result suggests a difference in the copy number of the rDNA-integrated p*TEF1*-SS-*AXH1*-Flag construction between the two recombinant strains.

### 3.7. Versatility of the Transformation/Expression Tools

While the different tools were applied in *B. raffinosifermentans*, we speculated if they were functional in another species of genus *Blastobotrys*. During our study on yeast biodiversity in the gut of insect larvae, we isolated a yeast strain belonging to a new species we named *Blastobotrys yvelinesensis,* closely related to *B. mokoenaii.* We thus used the protocol we newly developed for CBS 8335 to successfully transform this strain with the p*TDH3*-SS-V5-As*AXH1*-*PHO5*t expression vector.

Since *B. yvelinesensis* isolate L1-24 grows optimally at 30 °C, the recombinant clone was grown at 30 °C and samples were collected after 24, 48, 72 and 96 h of culture. The results depicted in Figure 5C showed that the *B. raffinosifermentans TDH3* promoter enables a quite high expression of Axh1 after 72 and 96 h of culture. While maximum levels of the recombinant protein were obtained after 48 h of growth for *B. raffinosifermentans*, 96 h of culture were required for the new species to accumulate significant amount of Axh1, a difference that may be explained either by the heterologous promoter or the lower temperature used.

### 3.8. A Xylanase Activity in B. yvelinesensis

**Figure 6:**
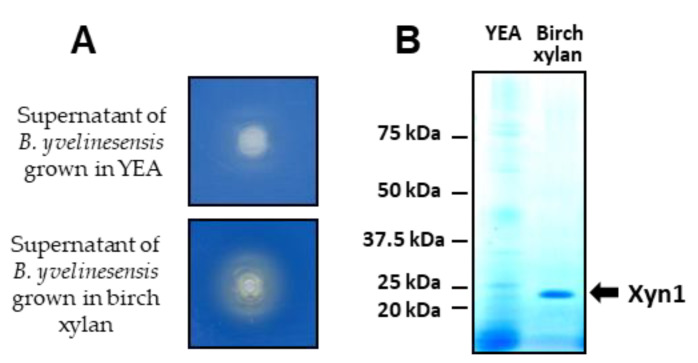
Detection of a xylanase activity in *B. yvelinesensis*. (**A**) Halo formation on RBB plates. Supernatants of *B. yvelinesensis* grown for 48 h at 30 °C in YEA with glucose (upper plate) or YEA with 1% birch xylan as sole carbon source (bottom plate) were filtered and concentrated before deposition in the central well. Plates were incubated for 24 h at 30 °C. (**B**) Concentrated supernatants were analysed by SDS-PAGE and total proteins were detected using EZ-blue reagent.

The new species is closely related to *B. malaysiensis*, *B. illinoisensis* and *B. mokoenaii* with 97% identity over 1139 nucleotides in the ITS and D1D2 domain of the rRNA gene (Table S4). The latter species is known to express a xylanase of the GH11 family. We therefore tested the presence of a xylanase activity in the culture medium of L1-24 grown on birch xylan. In contrast to cells grown on YEA, concentrated culture supernatant of *B. yvelinesensis* grown for 48 h in 1% birch xylan formed a halo area on a RBB xylan-containing solid medium after 24 h of incubation at 30 °C (Figure 6A). SDS-PAGE on concentrated samples of the two supernatants clearly revealed the presence of a band in the culture performed on 1% birch xylan that was not detected in the YEA culture (Figure 6B). The molecular weight of this protein was around 22 kDa, which corresponds to the expected size of the *B. mokoenaii* xylanase. These results suggest the presence of an endoxylanase in *B. yvelinesensis* L1-24.

**Figure 7:**
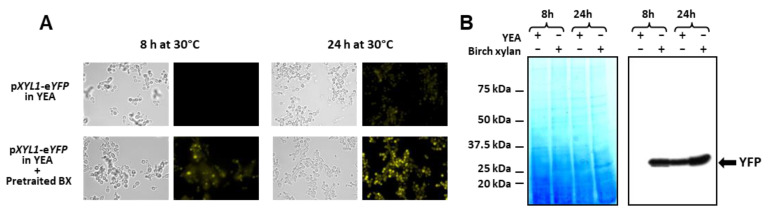
Induction of *XYL1* promoter. (A) YFP fluorescence visualisation. CBS 8335 cells expressing the YFP protein under the control of the *XYL1* promoter were observed after 8 and 24 h of growth at 30 °C either in YEA with glucose or with 1/10e (*v*/*v*) of birch xylan pre-incubated with *B. yvelinesensis*. (**B**) Detection of YFP. Intracellular protein extracts from samples collected for each condition: YEA with 1% glucose or YEA with 1% birch xylan, 8 h and 24 h, were analysed by SDS-PAGE and total proteins were detected using EZ-blue (left panel) or using monoclonal anti-FP antibody (right panel). Precision Plus Protein™ Standards(Bio-Rad) were used for gel calibration.

To confirm this hypothesis, the CBS 8335 strain containing the reporter YFP gene under the control of the *XYL1* promoter was grown in a YEA medium containing 1/10^e^ (*v*/*v*) of filtrated 1% birch xylan pre-incubated for 48 h with *B. yvelinesensis* instead of glucose. Fluorescence observations clearly showed induction of YFP expression after 8 and 24 h of incubation in the pre-treated xylan medium in comparison with glucose-containing medium (Figure 7A). This was confirmed by Western blotting (Figure 7B). Thus, some xylose oligomers were released during *B. yvelinesensis* growth on 1% birch xylan through expression of the endogenous endoxylanase and allowed induction of the *XYL1* promoter of the CBS recombinant clone inoculated in this pre-treated medium.

## 4. Discussion

The aim of this study was to provide new tools for high level constitutive or inducible gene expression in *B. raffinosifermentans* with the ultimate goal of developing sustainable fermentation processes by generating recombinant strains expressing glycoside hydrolases active on sugar polymers found in plant biomass. As only two promoters were available in expression platforms for this yeast, p*TEF1* and p*HSB4*, we characterised five new promoters, one of which was used for heterologous gene expression. Both *TEF1* and *HSB4* promoters belong to the family of constitutive promoters [18,38]. In our study, the *TDH3* promoter was shown to confer a higher expression of the downstream coding sequence than p*TEF1*. Similar results obtained by Xiong et al. showed the *TDH3* promoter in *S. cerevisiae* displayed its highest strength under both control and stress conditions [56]. In *B. raffinosifermentans* CBS 8335, we showed that expression under the control of the *TDH3* promoter is maintained for at least 72 h in a glucose, glycerol or ethanol containing medium and also at 42 °C, thus demonstrating the *TDH3* promoter to be a strong and stable constitutive promoter for heterologous expression likely to be of interest for industrial fermentation processes.

In addition, two inducible promoters were characterised; namely p*HSP26*, which is repressed at 30 °C and needs to switch to 42 °C to be induced, and p*PCK1*, which is repressed in glucose and needs a gluconeogenic carbon source such as glycerol to be induced. The *XYL1* promoter, which is early expressed in degraded xylan, could be useful for metabolic engineering of *Blastobotrys* for hemicellulose degradation. Moreover, a choice of inducible or repressive promoters is opportune when the protein synthesised confers some toxicity to the cells. The promoters described in this study thus offer a diversity of potential applications in the fields of heterologous protein production or yeast engineering.

Following the characterisation of these new promoters, the efficiency of two signal peptides, from the endogenous Abn1 and Agu1 proteins, was assessed through the secretion of a heterologous protein, namely Axh1 from *A. siamense*. Furthermore, the two expression and secretion vectors ensured the production of large amounts of proteins epitope-tagged either at their N-terminus with the V5 epitope or at their C-terminus with a Flag epitope. Both tags allow the purification of the recombinant protein. Until now, heterologous secretion of protein in LS3 was dependent on either the native signal peptide or the signal peptide of the *S. cerevisiae* α factor [3,38]. The expression and secretion of both endogenous and heterologous proteins under p*TEF1* or p*TDH3* were shown to be more efficient at elevated temperatures for the Abn1, Agu1 and Axh1 proteins. Previous results from Boër et al. on the heterologous expression of IL-6 under p*TEF1* showed that secretion was higher at 30 °C than at 45 °C, probably in line with the mycelial form of the LS3 cells [3]. Here, we used CBS 8335, which is still under a yeast form at 42 °C. This represents an additional advantage in biotechnological processes.

A rapid and efficient transformation protocol is a prerequisite for genetic engineering. Here, we developed a simple protocol based on lithium acetate and heat shock, as used routinely for other species such as *Yarrowia lipolytica*, and applied it successfully to the transformation of different *Blastobotrys* species [57]. Selection of recombinants was facilitated by the use of the dominant markers of resistance to hygromycin and nourseothricin, which enhanced possibilities for the introduction of multiple genes into a given strain. It is noteworthy we report the first use of nourseothricin in *Blastobotrys.* We also succeeded in targeting gene integration to specific loci, i.e., *TDH3* and *AGU1*, through homologous recombination, which also enhanced the activity of the *TDH3* promoter. This approach ensures that integration of the recombinant cassette does not impair expression at other loci and limits the copy number. Alternatively, rDNA targeting may lead to multiple integration events as observed for the p*TEF1*-controlled expression cassette and thereby improve the expression levels of the heterologous Axh1 protein.

The development of these new tools and the high-level expression of two glycoside hydrolases of the GH67 (Agu1) and GH43 AXH-like (Axh1) families paved the way for *B. raffinosifermentans* engineering for xylan utilisation. However, in order to hydrolyse the xylose polymer backbone, an endoxylanase is required. We identified a new *Blastobotrys* species that, as recently found for *B. mokoenaii*, was shown to secrete a xylanase. Further studies will thus consist of co-expressing this heterologous xylanase along with Agu1 and Axh1 to obtain *B*. *raffinosifermentans* strains capable of growing on xylan. This is expected, as this yeast possesses two endogenous xylosidases that may be expressed in the presence of xylo-oligomers. Indeed, the result obtained with the culture supernatant of *B. yvelinesensis* grown on birch xylan showed that the *XYL1* promoter is activated. We are thus confident that such engineered *B. raffinosifermentans* will have a set of xylan degrading enzymes efficient enough to hydrolyze sugar polymers found in various plant biomasses.

## Figures and Tables

**Table 1:** Selected promoters.

Gene Name	LS3 Gene	Size (pb) ^1^	Predicted Regulation
*HSP26*	ARAD1D12166g	460	Induced by heat shock
*PCK1*	ARAD1D32010g	1107	Repressed by glucose Induced by gluconeogenic substrate
*TDH3*	ARAD1D16896g	1153	Constitutive
*TEF1*	ARAD1C01892g	300	Constitutive
*TPS1*	ARAD1C43846g	636	Induced by osmotic stress
*XYL1*	ARAD1D50644g	879	Induced by xylobiose

^1^ Size corresponds to the intergenic region upstream of the CDS.

**Table 2:** Enzyme set for xylan degradation.

GH Family/CE	Name (EC Number)	LS3 Gene	Conserved Domains	Yeast Homologues (% Identity with LS3) ^1^
GH10/GH11	XYN1 (EC 3.2.1.8)	*-*	pfam00331	*S. stipitis* KAG2732737.1 (GH10*)* *Su. lignohabitans* XP_018736116.1 (GH10) *Su. lignohabitans* XP_018734939.1 (GH10) *T. ciferrii* KAA8917054.1 (GH10)
GH43	ABN1 (EC 3.2.1.99)	ARAD1D18216g	cd18831	*T. ciferrii* KAA8914121.1 (78%)
GH43	AXH1 (EC 3.2.1.55)	*-*	cd18619	-
GH67	AGU1 (EC 3.2.1.139)	ARAD1D23848g	pfam07488	*T. ciferrii* KAA8917114.1 *(*61*%)* and *T. ciferrii* KAA8917113.1 *(*67*%)*
GH3	XYL1 (EC 3.2.1.37)	ARAD1D50644g	PLN03080	*Su. lignohabitans* XP_018735137.1 (58%)
		CUT08920.1		*T. ciferrii* KAA8906613.1 (50%)
		ARAD1C05676g	PLN03080	*Su. lignohabitans* XP_018735137.1 (55%)
		CUT08919.1		*T. ciferrii* KAA8906613.1 (60%)
CE1	FAE (EC 3.2.1.73)	ARAD1A06094g	pfam07519	*-*
		ARAD1A19822g		-

^1^ S. *Scheffersomyces*; Su *Sugiyamaella*; T. *Trichomonoascus*.

**Table 3:** Enzyme set for xylose assimilation.

Name	CDS Used for BlastP	LS3 Homologue	Conserved Domains	Best Homologues to LS3 Protein (% Identity)
XR (Xylose reductase)	*S. stipitis* PICST_89614 (XP_001385181.1)	ARAD1C28094g	cd19115	*T. ciferrii* KAA8897533.1 (79%) *Su. lignohabitans* XP_018737989.1 (74%)
XDH (Xylitol dehydrogenase)	*S. stipitis* PICST_86924 (XP_001386982.1)	ARAD1D37840g (CAG34729.1)	-	*T. ciferrii* KAA8903833.1 (64%) *Su. lignohabitans* XP_018736469.1 (60%)
XK (Xylulo kinase)	*S. stipitis* PICST_68734 (XP_001387325.2)	ARAD1C08800g	cd07776	*T. ciferrii* KAA8897400.1 (59%) *Su. lignohabitans* XP_018737264.1 (59%)

## Data Availability

The whole-genome shotgun project of *Apiotrichum siamense* L8in5 was deposited at the NCBI under project PRJNA812413. The Illumina raw reads are available under SRA accession number SRR18212397. The D1D2 and ITS sequence of *B. illinoisensis* YB-1343, *B. malaysiensis* Y-6417, *B. mokoenaii* Y-27120, and *B. yvelinesensis* L1-24 and L2-36 can be found at accession numbers OM904992, OM904990, OM904991, OM904993 and OM904994, respectively.

## Supplementary Materials

The following supporting information can be downloaded at: https://www.mdpi.com/article/10.3390/jof8050418/s1, Figure S1: Cloning strategy for plasmids with different expression cassettes; Figure S2: Cloning strategy for the construction of secretion plasmids; Figure S3: Strategy for heterologous enzyme cloning and expression; Figure S4: EZ-blue gel corresponding to Figure 2A; Table S1: Yeast strains used in this study; Table S2: List of primers used in this study; Table S3: List of the plasmids used and constructed in this study; Table S4: Sequence identity in the ITS and D1D2 domains of the 26S rRNA gene of *Blastobotrys* species; File S1: Sequences and alignment of endo-1,5-alpha-L-arabinanases (GH43 AnAbnA-like); File S2: Sequences and alignment of alpha glucuronidases (GH67); File S3: Sequences and alignment of arabinoxylan alpha arabinofurano hydrolases (GH43 AXH-like); File S4: Sequences and alignment of beta-xylosidase (GH3); File S5: Sequences and alignment of feruloyl esterase.
