# Supplementary material for: Development of a Vector Set for High or Inducible Gene Expression and Protein Secretion in the Yeast Genus Blastobotrys
Source: J Fungi (Basel). 2022 Apr 19;8(5):418. doi: 10.3390/jof8050418 (PMC9144253; doi:10.3390/jof8050418)

Figure S1: Cloning strategy for plasmids with different expression cassettes

1- eYFP cloning at *Bam*HI-*Not*I sites of pBS-SA-p*TEF1*-*PHO5*t (Boër *et al.*, 2009)

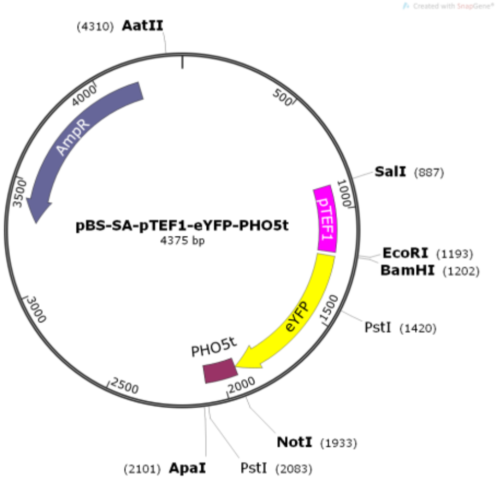

2- Promoter amplification and cloning at the *Sal*I-*Bam*HI sites of pBS-SA-p*TEF1*-eYFP-*PHO5*t

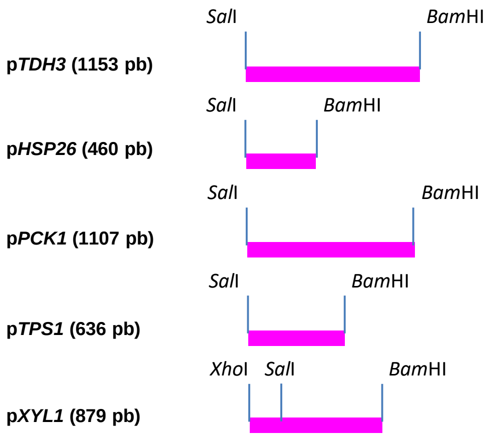

3- Cloning of expression cassettes at *Sal*I-*Apa*I the pARE12 plasmid (Sanaya *et al.*, 2020)

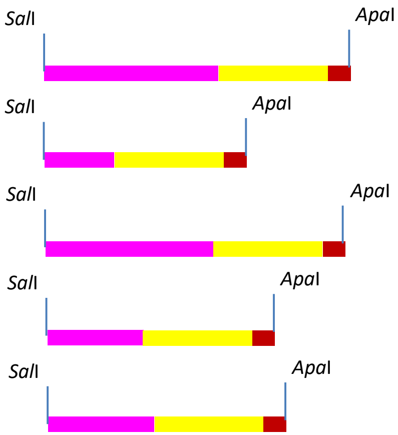

4-Cloning of the Hygromycin resistance cassette in the pBS-SA-p*TDH3*-eYFP-*PHO5*t at the *Aat*II-*Sal*I sites

The final pBS-SA-p*TDH3*-eYFP-*PHO5*t +Hph plasmid

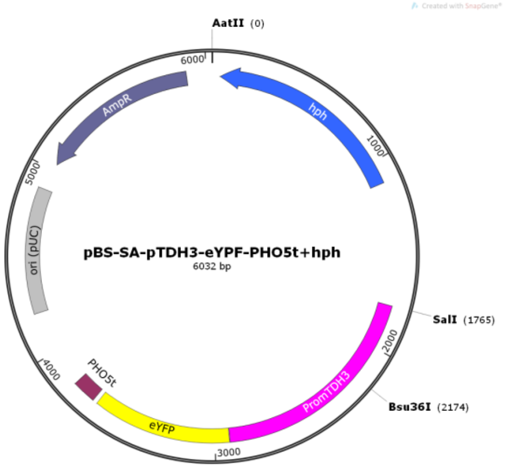

The 6 final pARE12 plasmids

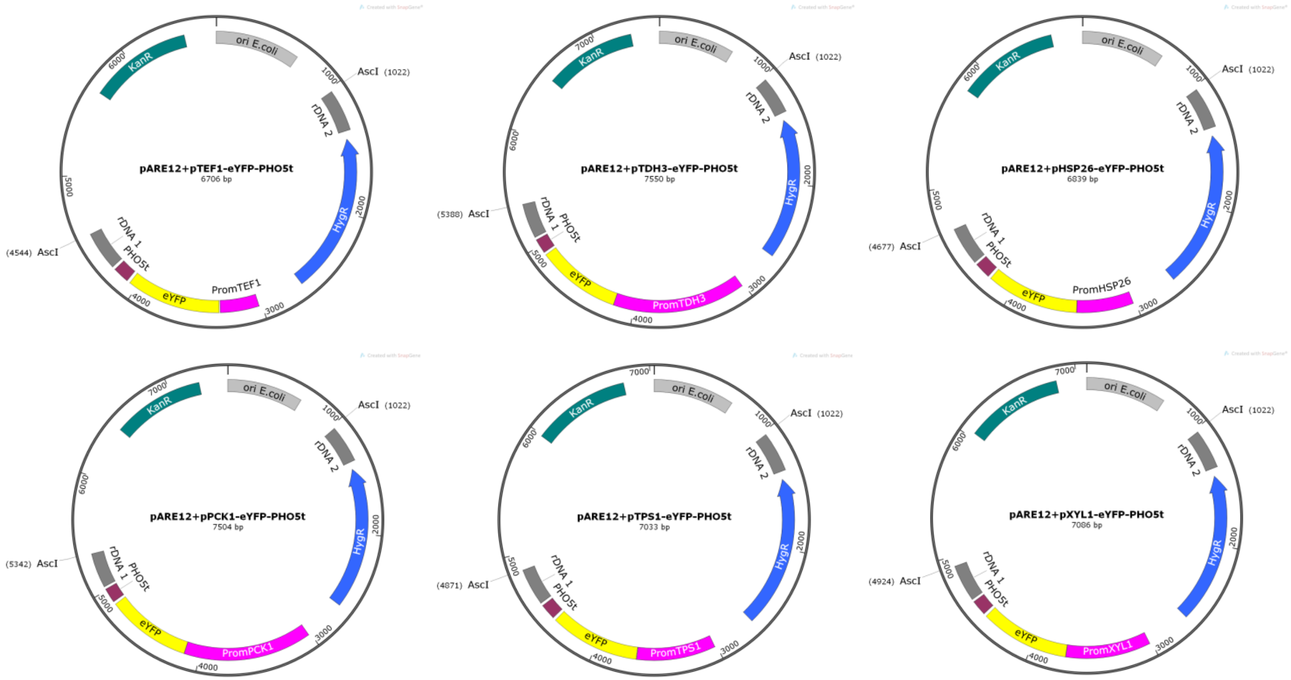

Supplement: Supplementary file 1 [file jof-08-00418-s001.zip › FigureS1.pdf]
