# Supplementary material for: Development of a Vector Set for High or Inducible Gene Expression and Protein Secretion in the Yeast Genus Blastobotrys
Source: J Fungi (Basel). 2022 Apr 19;8(5):418. doi: 10.3390/jof8050418 (PMC9144253; doi:10.3390/jof8050418)

Figure S2: Cloning strategy for the construction of secretion plasmids

A

1- V5-*BrABN1* cloning at *Bam*HI-*Not*I sites of pBS-SA-p*TDH3-eYFP-PHO5*t (Figure S1)

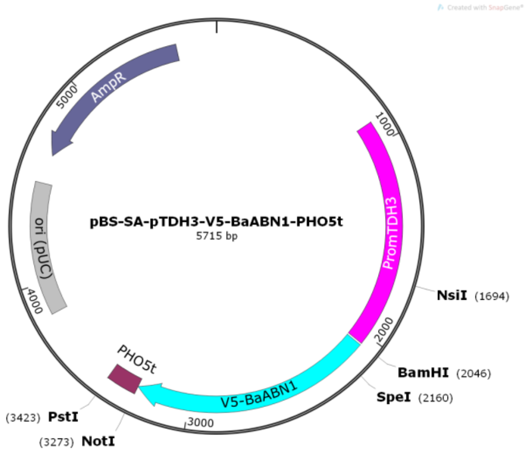

2- nat cloning at the *Pst*I site of pBS-SA-p*TDH3*-V5-*BrABN1-PHO5*t

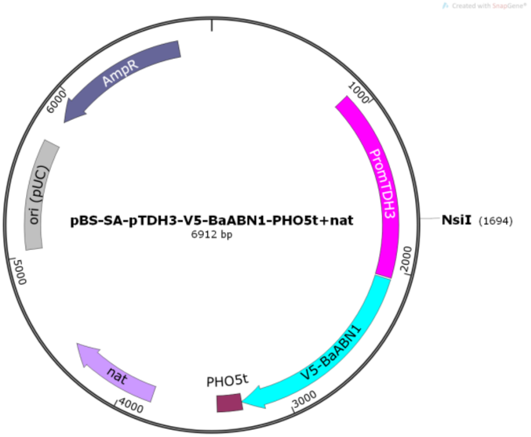

B

1- 6xHIS-*BrAGU1* cloning at *Bam*HI-*Not*I sites of pBS-SA-p*TEF1-eYFP-PHO5*t (Figure S1)

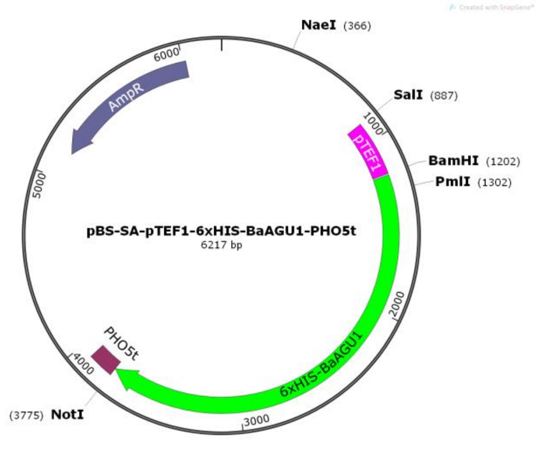

2- hph cloning at *Nae*I-*Sal*I site of pBS-SA-p*TEF1*-6xHIS-*BrAGU1-PHO5*t

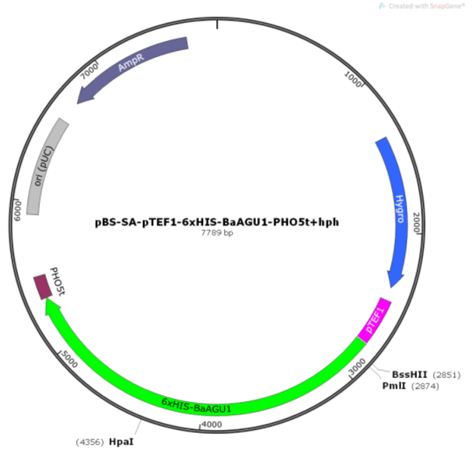

Supplement: Supplementary file 1 [file jof-08-00418-s001.zip › FigureS2.pdf]
