# Supplementary material for: Development of a Vector Set for High or Inducible Gene Expression and Protein Secretion in the Yeast Genus Blastobotrys
Source: J Fungi (Basel). 2022 Apr 19;8(5):418. doi: 10.3390/jof8050418 (PMC9144253; doi:10.3390/jof8050418)

# Figure S3: Strategy for heterologous enzyme cloning and expression

**A**

**1- *AsAXH1* cloning at *Spe*I-*Not*I sites of pBS-SA-p*TDH3*-V5-*BrABN1*-*PHO5*t (Figure S2A)**

**2- nat cloning at the *Pst*I site of pBS-SA-p*TDH3*-SS-V5-*AsAXH1*-FLAG-*PHO5*t**

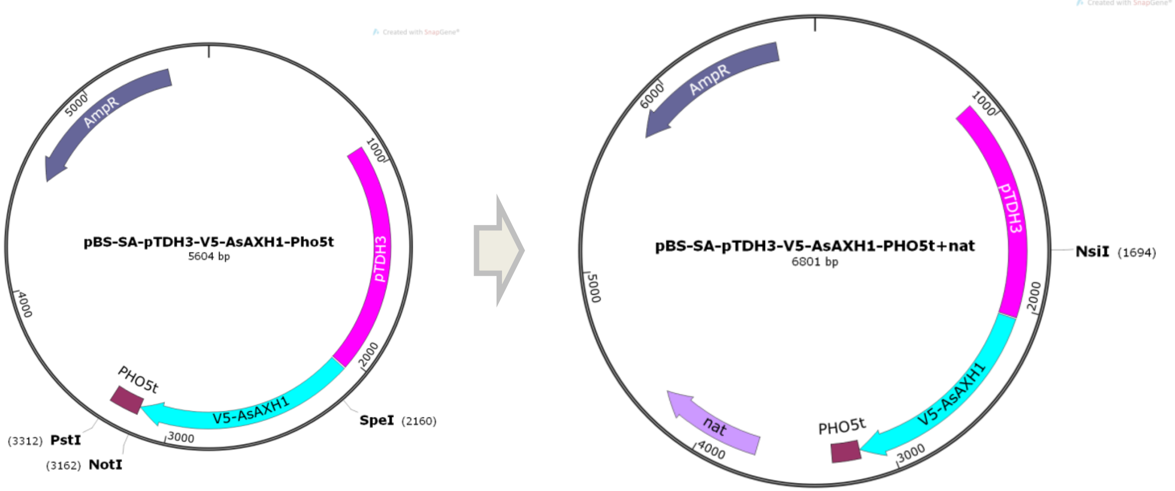


**B**

**1- *AsAXH1* cloning at *BssH*II-*Not*I sites of pBS-SA-p*TEF1*-6xHIS-*BrAGU1*-*PHO5*t (Figure S2B)**

**2- nat cloning at the *Pst*I site of pBS-SA-p*TEF1*-SS-*AsAXH1*-FLAG-*PHO5*t**

**3- LS3 rDNA cloning at the *EcoR*V site of pBS-SA-p*TEF1*-SS-*AsAXH1*-FLAG-*PHO5*t +nat**

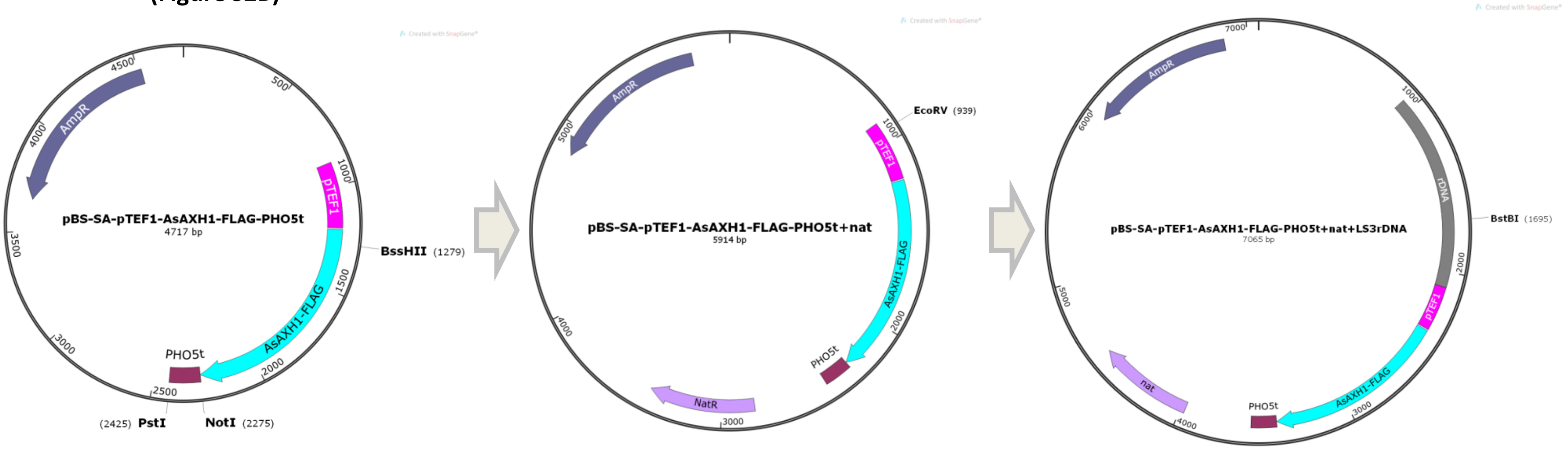

Supplement: Supplementary file 1 [file jof-08-00418-s001.zip › FigureS3.pdf]
