# Supplementary material for: Development of a Vector Set for High or Inducible Gene Expression and Protein Secretion in the Yeast Genus Blastobotrys
Source: J Fungi (Basel). 2022 Apr 19;8(5):418. doi: 10.3390/jof8050418 (PMC9144253; doi:10.3390/jof8050418)
Supplement: Supplementary file 1 [file jof-08-00418-s001.zip › FileS1.pdf]

File S1: Sequences and alignment of endo-1,5-alpha-L-arabinanases (GH43 AnAbnA-like)

```
>Aspergillus.niger_CBS513.88_XP_001397586.2 arabinan endo-1,5-alpha-L-arabinosidase
D
MKSITMLPSLLSVLFFLLYAVNAVPLAPRASALAGIDTKSFSKTKDYPLPNLGNIVAHDPNVIQHDGYFY
LYKGGVHIPIHRARSLSGPWEQVGTVLDDSSVIPKQNRSRPWAPTTIQHDNRFYCFYAISENGSRDSAIG
VASSDTPVGGNWTDHGAVVNTGKGDLSDIYPYSVSNAIDGAFITDQQTGQSHLLYGSYWHGIFSVPLADD
LLSVKTPKTPNATNLAYIPDAKSKPIEGSFMTYKAPYYYLWFSHGKCCHFDIHAFPPMGDEYNIRVGRSK
SATGPFVDKDGHDTLKGGGTIVYGSNHGIVYAPGGVGVLINNGSEADVLYYHYLNTTSGFAQGDAHLGWN
YLHYVNGWPVAVEGYVNANGK

>Blastobotrys.raffinosifermentans_ARAD1D18216
MKSQLIATIATVALGSMSSVVAAKSKTDDSNTYDRFRGLVDSNTYDLMNNSLGGTYVHTPNLTVIDTPD
WPLPNPVIGDIVPWGGVHLHDPSIIKHNGYYYSFTTHNLIGISKAPSMFGPWQKIGSVLEESSIINSTG
STDPWAPDVQKVGDTFYCYYAVSSFGSPKSSIGLATSKTLEPGSWTDHGEVISSGPNAPYPLNDSNAID
ANLLVVENGNSVQEAYLLWGSFWSNIWQIKLNNDLTVPDNAIANAVQLAYDGTIDTHPVEGAYLHKASN
GYYYLFVSNGVCCGYDAALPDAGREYKILVGRSKSPSGPFLDKNGVDMAKGGGSRIYGSHGIVYGPGGQ
GVFTDDDGQDIIYYHYVDLRVSLADDDKRLGWNYLKYEDGWPKLV

>Blastobotrys.mokoenaii_ABN1
MKVILQTIAVSLLATLSAAADSSSDQVNRFRGIVDQSVYNTLNNSLGGTYVHTPNFTVIDTPDWPLPNP
VVGDIVPWGGVHIHDPSIIKHNGNYYSFSTHDLVAISKAPTMYGPWTKIGSVLEKQSIIENSGRGDPWA
PDVHKVGDTFYCYYAVSSFGSQNSSIGLATSKTLEPGSWTDHGAVLTSSPSAEHPLDVSNAIDANLFVV
EDKKGNVQDAYLLWGSFWSNIWQIKLNKDLTVPDGALEKAVNVAFDGTIDTHPVEGAYLHLAKNGYYYL
FVSNGACCGYSNGLPDAGREYKILVGRSKSPSGPFLDKNGKSMAEGGGYRIYGSHGNIYGPGGQGVFTD
DDGQDIIYYHYVDLRVSLVDEDKRLGWNYLKYDKDGWPYLA

>Trichomonascus.ciferrii_KAA8914121.1
MRFFLIACFWGAAAVAKESVEPSTYDLLNNSVGGTYVRESQFTVANTPDWPLPKPVTGDTDPWGGVHVHD
PCILKHDGYYYSFTTHNLVGISRAKSMEGPWEKIGSVLEGSSIINNTGSADPWAPDVHKVGDTFYCYYSV
STFGSQNSSIGLATSKTLEPGSWTDHGPVLTSTPDAQFPLNDSNAIDANLMVVEKKNEVKEAYLLWGSFW
SNIWGVKLNEDLSMPEDALDNAIHYAYDSTIDTHPVEGPYLHHARNGYYYLFVSNGVCCGYNNSLPDAGR
EYKILVGRSKNPEGPFLDKNGIDMVNGGGSRIYGSHGIVYGPGGQGVFTDDNGEDIIYYHYVDLRVSLID
EDKRLGWNYLKYDKDDWPYLV

>Tothia.fuscella_KAF2437171.1
MFALTTIWPLVIAIASALTTSNPFSQSNWPLPHNITGTDTPGQWGGVHLHDPSIVQGPDGAYYSFSTHGL
VSISRAVSLDGYWEHIGSVLTRQSIIDLPGRNDTWAPDVSKIGNTYYCYYSISTFGSQNSAIGLATSQTL
LPGSWTDHGAVLESGKDKRYPLNATNGIDANIFVDEKSGEVFLNYGSFWSDIWQFKLLKDLKGLDWSWPP
KQLSFEPKGTNPEEGASISENKGWYYLWVSHGVCCGFNTTLPVKGEEYEILVGRSKTHYGPFVDREGKDM
KNGGGSVVYGSHEYVYGPGGQGVLKNYNGRDVLYFHYVPTYVSYIDEDKRLGWDYIEYVDGWPVLVY

>Rhizodiscina.lignyota_KAF2093984.1
MLLLVFALIAIAARAASHDPFSPANWPLPHNITGDSLTSTGQWGGVHVHDPSVVKGPDGYYYSFSTHNLV
AISKAPSLDGYWKFIGSVLKGESVIPLAGRNDTWAPNVVQIGDTYYCYYSVSTFGSMDSGIGLATSRTLQ
PGSWTDHGLVLESGATKEYPLNDTNAIDPNFFHDPKTGENLLNYGSFWSDIWQFQLAKDLKSVLWKPPPV
HLSFDPVGTNPEEGSFMSYHDGWYYLWYSHGICCGYNVSALPPPGQEYSIRLGRSKSSHGPFLDRNGTDL
VDGGGYIVYGSHDYVYGPGGQGVLQDYHGRDVLYYHYVPTYISYLDTDKRLGWDYIDYVDGWPVLVY

>Aureobasidium.pullulans_THY28006.1
MYALITKIGALAVASLATQAVAVPSNSKPDPFSNASWPLPHNVSGVSNPGANESYQGWGGVHLHDPSIVM
GPDGHFYSFSTHGLVVISRASQPNSLDGYWEPIGSVLEGSSIINNTGSTDPWAPDVHKVGDTYYCYYAVS
QFGSQFSSIGLATSKTLRPGSWVDHGAVLTSNTTAPSPLNITNAIDANLFIDPKTKIPYLTYGSFWSDIW
QFQLAKDLLSVVWTPAPVQLSLDPVSPQAEEGAYLSRHDSWYYLWYSHGSCCGYNVSALPAPGQEYSIRV
GRSRSATGPFVDRNGVDLRNGGGYIIYGSHDYVYGPGGQAVLTNYHGRDVLYYHYVNTIQNPTYVDELKL
LGWNYINYIQGWPVLSYD

>Hortaea.werneckii_RMX89073.1
MPCFNGFTALALASLGTSVSTVLSAPSGDWKGHGGWRGHKDPFSNASWPLPRKVYGENDTANIDSATEGW
GGIHLHDPSIVLGPDGHYYSFSTHGLTVISRASKKGSIDGYWEIIGSVLDAGSSVIDNSGSTDPWAPDVH
KVGDTYYDYYSVSQFGSQISAIGLATSKTLMPGSWTDHGEVFSSDPNATYPLNVTNAIDANLFIDPKTKT
PYINYGSFWSNIYQFELTDDLKDVKQDTALQLSLDPVAPQPEEGAYMNYHDGWYYLWYSHGTCCGYNASA
LPAPGEEYSIRVGRSRSPRGPFVDREGVKLTEGGGYIVYGSHDFVYGPGGQGVLSDYHGRDVLYYHYVNT
IVDPSYADNLKLLGWNYIDYIQGWPVLSYH
```

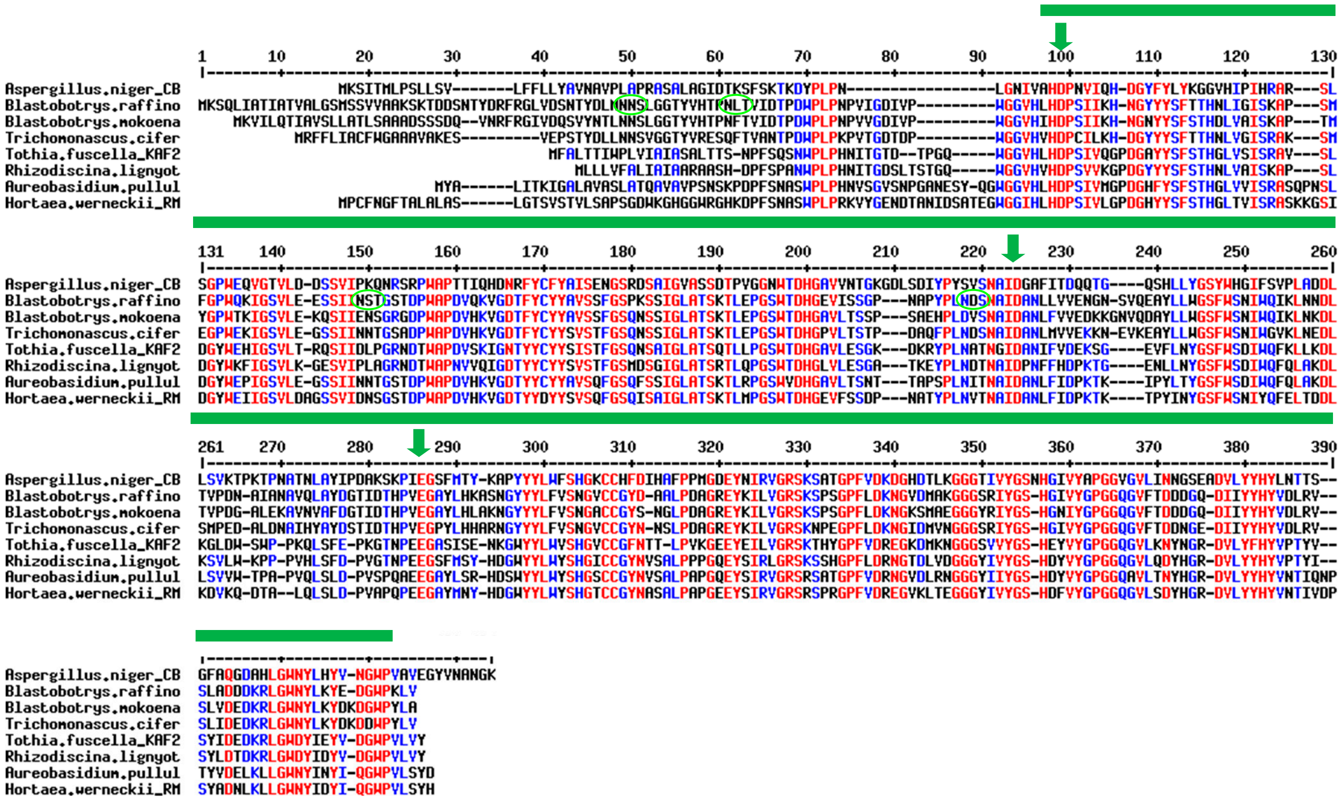

Alignments were performed with the Multalin server (http://multalin.toulouse.inra.fr/multalin/). The *B. raffinosifermentans* ARAD1D18216 predicted protein was aligned with *Aspergillus niger* CBS513.88 XP_001397586.2, *Blastobotrys mokoenaii* Abn1, *Trichomonascus ciferrii* KAA8914121.1, *Tothia fuscella* KAF2437171.1, *Rhizodiscina lignyota* KAF2093984.1, *Aureobasidium pullulans* THY28006.1 and *Hortaea werneckii* RMX89073.1 conserved proteins

Amino acids 104 to 402 of the *B. raffinosifermentans* Abn1 protein correspond to the cd18831 conserved domain characterising the *Aspergillus niger* endo-alpha-L-arabinanase (AbnA)

Amino acids that form the active site in the conserved domain cd18831: D at position 90, D at position 207 and E at position 267 for the *B. raffinosifermentans* Abn1 protein.

N-glycosylation sites predicted in *B. raffinosifermentans* Abn1 protein: N at positions 49, 61, 135 and 201.
