# Supplementary material for: Development of a Vector Set for High or Inducible Gene Expression and Protein Secretion in the Yeast Genus Blastobotrys
Source: J Fungi (Basel). 2022 Apr 19;8(5):418. doi: 10.3390/jof8050418 (PMC9144253; doi:10.3390/jof8050418)
Supplement: Supplementary file 1 [file jof-08-00418-s001.zip › FileS2.pdf]

File S2: Sequences and alignment of alpha glucuronidases (GH67)

>Blastobotrys.raffinosifermentans_ARAD1D23848
MCIDFTMQFLLPLVSLGFLGLAAAENGIDGWLRYAPLPCNHQCKSELPGSIVALNSTQSSPIYVAGAELQKGLKSIYGQQADVSHDKCDSSSSSVVIGTVAQYDRSCGPVKDA
IPQLDADGFWIDTRGDQIRILGSNERGALYGTFEYLSMIAQGNMSKVAYATNPSAPLRWTNEWDNMDGSIERGYGGSSIFFANGTVVEDLTRVQQYGRLLASLRINTVVINNV
NANASLLTSENIKGLGRVADTFRPWGIQVAAALNFASPKTVGGLDTFDPLDDSVIKFWSNITDSIYTQVPDFAGYLVKASSEGQPGPSTYNRTLSDAANLFANALEPYGGVVM
FRAFVYDQLNESNWDADRAKAAVDYFKPLDGKFKDNVVVQIKYGPIDFQVREPASPLFANLPNTNTAIELEITQEYLGQQSHLVYLAPLWKELLDFDLRADNKSSKVQDVVTG
KRFNRLLGGFAGVVNVGSNSTWLGSHLSMSNLYAYGRLAWNPADDPRSILQDWIRLTFGMDEKVVNVITQMSMESWPAYENYSGNLGIQTLTDILYTHYGPNPASQDNNGWGQ
WTRADHNTIGMDRTVSNGTGYSGQYPSEIAAMYENIETTPDNLLLWFHHVNYTQPLKSGKTVIQHFYDAHYDGAETANSFPAMWQTLKGKIDDERYDHVLHRLEYQAGHSLVW
RDAIVNFYHNLSGIGDTSKRVGYHPWRIEAEDMTLDGYKPYTVSPFETASGSVAIVTSTNTTVGTASTHVNFPSGKYDLAINYYDLYGGQSQWKVYLNDKKIGEWQGNMENVL
SHNPSIYLDGHSATRIKFKDVNVKKGDVLKIVGTPDGIEPAPLDYVAFLPGGIID

>Trichomonascus.ciferrii_KAA8917114.1+KAA8917113.1
MNLPNEIVALNHSETSPVFTAGNEVLRGLEGIFDHHASVKHDGCGSRSNVVIGTAHAFKSSCTGVIVPEIEEGFWLSINGSSVKIVGQNDRGALYGAFEYLGMMAQGNFTAVD
YVTNPDQPIRWVNQWDNFDGTIERGYEGPSIFFANYSIVDDLGRANEYARLLASIRVNGIVVNNVNANETLLKDENIIGLARIADVFRPWGIPIGISLNFAAPKDLGGLETFD
PLDQKVIQFWQDRSDTIYKLIPDLAGYLVKGDSEGQEGPLTYNRTLADGANLFANAIKKYGGIVMYRAFVYEKLNKTDWKSDRAKAAYDYFQPLDGKFEDNVVVQVKYGPIDF
QVREAISPLFGSLRHTNVAVELQITQEYLGQQCHLVYLPPLWRTVWDFDLRTDNESTLVSEVVSGRRFNRPLAGSAAVVNVGTNDT::MIDAITTMSMNSWSAYEKYSGNLGI
QTLADILYTHFGPNPQSMDNNGWGQWTRSFHETVGMDRTVENGTGYTGQYPPEVAALYEDVTTTPDDLLLWFHHVPYTHRPKSSNKTVIQHFYDSHYEGAEMANEFLTLWESL
EGKIDTQRYHETLFRQKYQAGHSIVWRDAINNFYNNISGILDEAGRVGRHPWRIEAEEMHLDGYELYTVSPFEMASNSTAIVTSSNATSGTASIIIPFEDGKYDIAIGYYDLF
DGKAQWEATINNKQLGSWTGDNEDHLGHEQSRYLDGHTATRITFRNIKVNNGDELKVKGTPNGIEPAPLDYVAFLPNGIID

>Penicillium.roqueforti_XP_038928484.1 CAZyme family GH67
MRTLLFFVSLGFAAAENGLNGWLRYASLPCSGQCHYNLPSSISILNATETSPVYVAGTELQNGLKGIYGKSVQVTHNCNISSVVVGTVDQYRKSCGAVNSVPELEEDGFWLDT
KGGNVQILGQNERGALYGTFEYLSMLAQGNFSKVAYATNPSAPIRWVNQWDNMDGRIERGYGGPSIFFKDGQIVDDLTRVAGYARLLASIRINAVVINNVNADATLLNPTNLD
GVARIADVFRPYGIQVGLSLNFASPQTYGGLSTFDPLDTSVIAWWSNITTQIYDLVPDMAGYLVKADSEGQPGPQTYNRTLADAANLFAKEVQPYGGIVMYRAFVYNQLNESV
WTDDRAKAAVEFFKDLDGEFDDNVVIQIKYGPIDFQIREPASALFANLFNTSMAIELQVTQEYLGQQSHLVYIAPLWKTILDTDLRVDHQPSLVRDIVAGKRFNRRLGGSAAV
VNVGTNTTWLGSHLSMSNLYAYGRLAWNPADDAQDILQDWIRLTFGLDRKVLGTITRMSMESWPAYEQYSGNLGIQTLTDILYTHYGPSPASQDNNGWGQWTRADQTSIGMDR
TVANGTGFSGQYPDEIAAMYENIETTPDDLLLWFHHVKYTHRLHSGKTVIQHFYDEHYRGAETAQTFLTQWKSLHGKIDTERYNHVRHFLDYQSGHSIVWRDAINDFYYNLSG
IPDESKRAGHHPWRIEAEDMELDGYEAYTVSPFETASGSVAIVTTSNNTAGTASTKIKFPSGTYDLAVNYYDVYGGQSRWKIYLNDHEIGQWVGNSEDVLSHTPSIYLDGHSA
IRIKFRGVKIHKGNTLKIVGMPNGTEFAPLDYVALLPAGIVD

>Rasamsonia.emersonii SAL89189.1 Glucuronidase
MRRLLLLASAALVAAENGLDGWLRYAPLPGASRYLHDLPSSIVSLNASETSPVYVAGSELQKGLQGIFGKQVSISHQNCNASSSIVVGTVDEYSKLCGSSAALPELEQDGFWL
STKGNSVQILGRNERGALYGAFEYLSMLAQGNFSQVAYANNPRAPIRWVNQWDNLDGSIERGYGGASIFFENGGVVSNLTRAAQYARLLASIGLNAVVVNNVNANASILTPQN
IEGLGRIADVFRPYGVQLALSLNFASPTIIGGLSTYDPLDPSVIAWWTNVTDQLYQRIPDFAGYLVKADSEGQPGPLTYNRTLAQGANLFAKPLQPYGGIVMFRAFVYNQLNE
SDWKADRAKAAVEFFQGLDGQFDDNVVVQIKYGPIDFQVREPTSPLFAHLRNTSTAIELEITQEYLGQQCHLVYLPPLWKTVLDFDLRVDQKPSLVRDIITGQRFNRTLGGSA
GVVNVGTNTTWLGSHLAMSNLYAYGRLAWDPANDPQSVLQDWIRLTFGLDRSVIDTITQMSMESWPAYENYSGNLGIQTLTDILYTHFGPNPASQDNNGWGQWTRADHTSIGM
DRTVWNGTGYTGQYPPEVAAMYENIDTTPDNLVLWFHHVNYTHRLHSGKTVIQHFYDAHYSGAETAQTFLTMWESLKGKIDDERYNDVHFRLTYQAGHSIVWRDAINNFYFNL
SGIPDEAKRVGNHPWRVEAESMTLSGYKTYAVNPFETASNTTAIVTTTNSTVGTATTKLEYPSGTYDLAVNYYDMYGGKSHWQVYLNNRKIGEWVGNSEDTLGHTPSIYLDGH
SATRITFRGVKIEKGDELKIVGTPDGVEPAPLDYVSVLPEGVVD

>Aspergillus.fumigatus Af293_XP_753219.1 alpha-glucuronidase
MWSGIPIFALLSSIGIAAAETGLDGWLRYASVPCNGNCQRALPSHIVTLNSTRSSPVYVAGQELQDGLHQILGKHASVKSTGCSTDSSIIVGTVEAYRQVCNAGRQVPQFDVD
GFWLSIREKSVLIVGQSERGALYGAYEYLSMLAQGNFSQVSYATSPHAPIRWVNQWDNMDGSIERGYGGPSIFFKDGVIRQDLSRVQQYARLLASVRINGIIVNNVNANASLL
MPSNMDGLARIADIFRPYGIRVGISLNFASPSTLGNLSTYDPFDSSVIAWWGNVTDQLYARIPDMAGYLVKANSEGQPGPTTYNRTLADGANMFARALKPYGGVVMFRAFVYD
HHISEDNWYNDRANAAVDFFKPLDGKFDDNVVVQIKYGPIDFQVREPASPLFANLYKTNTAIELQVTQEYLGQQSHLVYLPPLWQTILGFDLRVDQKPSLVRDIISGQRFDRP
LGGWAAVVNVGTNSTWLGSHLAMSNLYAYGRLAWEPTLDSEDIVQDWIRLTFGLDRRIVDTLTQMSMESWPAYENYSGNLGIQTLTDILYTHYGPNPASQDGNGWGQWTRADH
LSIGMDRTVKNGTKFSGQYPAEVAAMYENIETTPDNLLLWFHHVNYTQRLHSGKTVIQHFYDAHYTGAETAQTFVSQWESLRERIDAERYQHVLTRLIYQAGHSIVWRDAINN
FYHNLSGIADEKQRVGHHPWRVEAEDMQLDGYVPYAVSPFETASNYTAIVTASNGTTGTASATLDFKTGTYDLGINYYDMYGGKSHWTVYLNDRVVGQWQGNSEDVLSHTPSI
YLDGHSATRITFRDVKIHKGDRLKIVGKPDGVEPAPLDYVVVLPPGIVD

>Aspergillus.fischeri NRRL 181_XP_001259234.1 alpha-glucuronidase
MWSGIPVFALLSSIGIAAAENGLDGWLRYASVPCNGNCQRALPSHIVTLNSTKSSQVYVAGQELQDGLHQILGKHASVKSTGCSTDSSIVVGTVEAYRQVCNTGSQAPELDVD
GFWLSIRGKSVQIVGQTERGALYGAYEYLSMLAQGNFSQVSYATSPHAPIRWVNQWDNMDGSIERGYGGPSIFFKDGVIPQDLSRVKQYARLLASVRINGIVVNNVNANASLL
MPSNMDGLARIADVFRPYGIRVGISLNFASPSTLGNLSTYDPFDSSVIAWWGNVTDQLYARIPDMAGYLVKANSEGQPGPTTYNRTLADGANMFARALKPHGGVVMFRAFVYD
HHISEDNWYNDRANAAVDFFKPLDGKFDDNVVVQIKYGPIDFQVREPASPLFANLYKTNTAIELQVTQEYLGQQSHLVYLPPLWQTILRFDLRVDQKPSPVRDIISGQRFDRP
LGGWAAVVNVGTNTTWLGSHLAMSNLYAYGRLAWEPTLDSEDIVQDWIRLTFGLDRRVLDTLTQMSMESWPAYENYSGNLGIQTLTDILYTHYGPNPASQDGNGWGQWTRADH
LSIGMDRTVKNGTKFSGQYPAEVAAMYENIETTPDNLMLWFHHVNYTQRLHSGKTVIQHFYDAHYDGAETAQTFVSRWESLRERIDAERYQHVLARLIYQAGHSIVWRDAINN
FYRNLSGIADEKERVGYHPWRVEAEDMQLDGYVPYAVSPFETASNFTAIVTASNGTTGTASATLDFKTGTYDLGINYYDMYGGKSHWTVYLNDRVVGQWQGNSEDVLSHTPSI
YLDGHSATRITFRDVKIHKGDRLKIVGEPDGVEPAPLDYAVVLPRGIVD

>Coniochaeta.ligniaria NRRL 30616_OIW35663.1 putative alpha-glucuronidase A
MRNILLVLLSAGLAAAEDGLSGWLRYAPLSCGRSSHLSLPQTIVALNTTISSPVYTAGQELQKGLQGIFAKQVKISDQTTKSSSVVVGSLAQYIKATGNSDVPSLEEDGYWLS
VKGDSVQIVGQNERGALYGAFEYLSMLAQGNFADVAKVSNPSAPIRWTNEWDNMDGSIERGFAGPSIFFANGVVVDDLTRVAEYARLLASIGINGIIINNVNANATTLSTQNI
RGLGRVADAMRPYGVQIGISLNFASPQSFGGLSTFDPLDASVIKFWTDITNQIYQQVPDFAGYLVKANSEGQPGPITYNRTLADGANLFAKAIKPHGGIVMFRAFVYDQLNES
DWHADRADAAVNYFKPLDGEFDDNVVVQIKYGPIDFQVREPASPLFANIPKTNTAIELQVTQEYLGQQCHLVYLPPLWKTVLDFDLKVDGQPSLVRDIVSGERFKRPLGGYAG
VVNVGTNSTWLGSHLAMSNLYAYGRLAWDPTSDAQAILQDWIRLTFGLNRIVVDTITKMSMDSWPAYENYSGNLGIQTLTDILYTHFGPNPASQDDNGWGQWTRADHNGLGMD
RTVSNGTGFAGQYPAEVAAVYESLEITPDNLLLWFHHVPYTYTLHEGKTVIQHFYDAHYSGAETAQTFPTLWEGLKGKIDDQRYEEVLFRLKYQAGHSIVWRDAISEFYFNKS
SIPDQANRVGNHPWRVEAESMQLSGYKVTAVNPFETASNFKAVVTSSNTTAGTATAKLNFPSGTYDLAINYYDIIGGRARYEVYLNDKLVGKWTGDNEDKLGHTPSTFLDGHS
ATRITFGGVKVETGDTLKIVGTPDGTELAPLDYVAVLPSGVID

>Aureobasidium.pullulans_AAR87862.1 alpha glucuronidase
MQLLTLFALFGLALAETGLDGWLRYAALPSSISWPSVPKHIVVLNTTETSPVYTAGQELQRGIQSILGQNCRFSTNKPDESIIVGTLDAYVAAYGNFSQSVNLKEDGFWLSTE
NNTVQIVGQNERGALYGAFEYLSMLAQANFSTVAYVSNPDAPIRWVNQWDNLDGSIERGYGGASIFFANGTIVNDLTRVAEYARLLASVGINAIVINNVNANASILSPQNIDG
VGRIADTMRPYGVQIGLSLYFASPTSGVQGQANLTTFDPLDPGVVTWWGNVTDQIYERIPDMAGYLVKANSEGQPGPITYNRTLAEGANLFARALQPHGGRVLFRAFVYNQLN
ESDWRADRANAAVDFFKPLDGKFDDNVVVQIKYGPIDFQVREPASPLFANLFHTNTAIELEVTQEYLGQQTHLVYLPPLWDTVLGFDMRIDNETSLVRDIIAGRTFNRSLGGY
AAVVNVGTNQTWLGSHLSMSNFYAYGRLAWNPTEDTTKIHEDWTRLTFGLDQNVVDTITHMALESWPAYENYSGNLGIQTLTDILYTHFGPNPQSQDNNGWGQWTRADHTTIG
MDRTVSNGTGYSGTYPPEVAAMYENIETTPDNLLLWFHHVNYTQKLHSGKTVIQHFYDAHYAGADTAQTFPEQWQTLAGKIDSQRFDEQLYRLKYQAGHSLVWRDAIVNFYHN
ISGIADENGRVGNHPYRIEAESMTLNGYEPYAVSPFETASDYYAVVTSDNSTTGSVSTRLHFPAGTYDVGVNFYDMYGGISKYEMLINNKTVATWAGDSENYLGHTPSIYLDG
HSARRITFTDVTVKEGDTLEIVGIPNGIEPAPLDYVVFLPKGMID

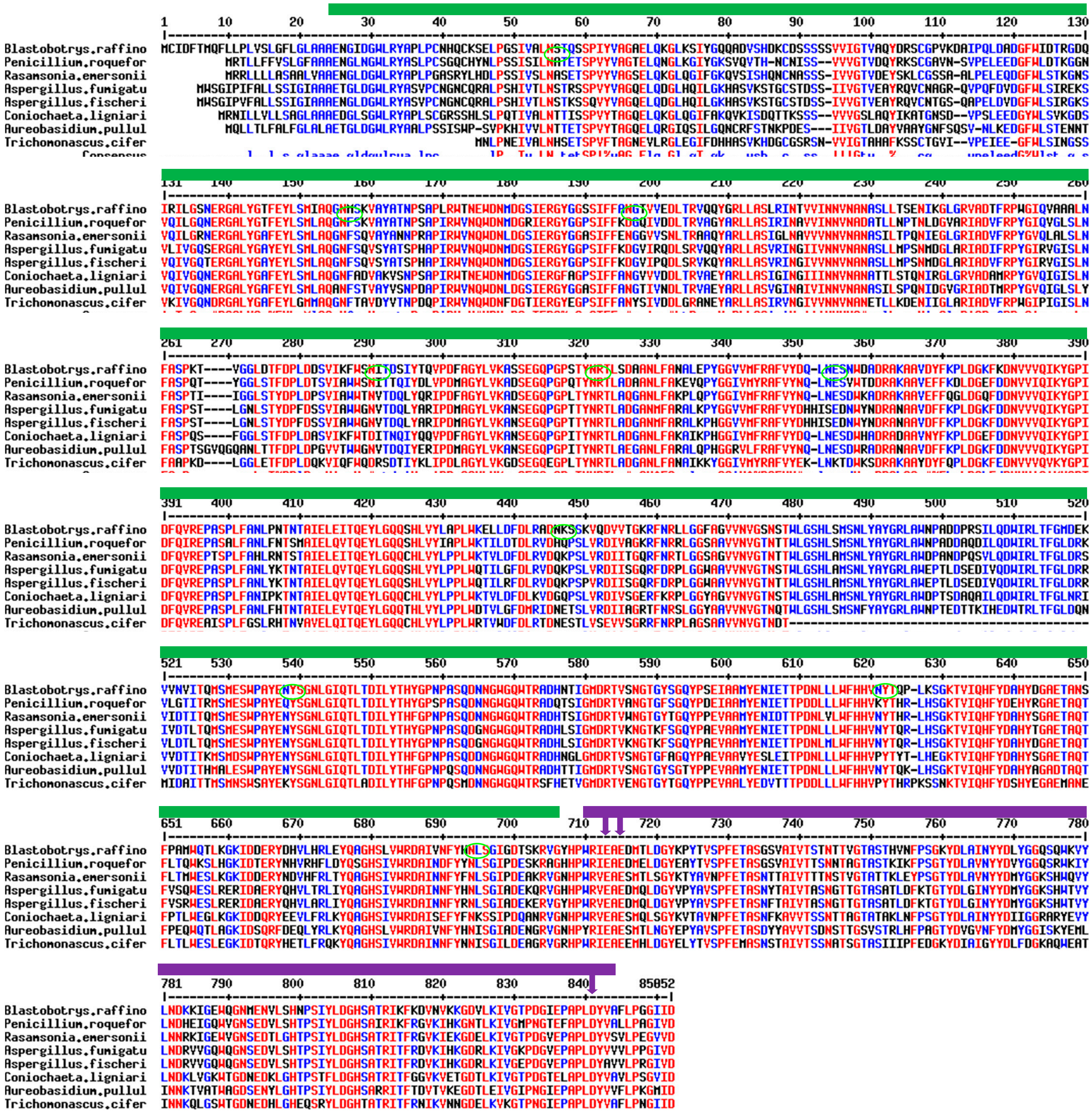

Alignments were performed with the Multalin server (http://multalin.toulouse.inra.fr/multalin/). The *B. raffinosifermentans* ARAD1D23848 predicted protein was aligned with *Penicillium roqueforti* XP_038928484.1, *Rasamsonia emersonii* SAL89189.1, *Aspergillus fumigatus* Af293 XP_753219.1, *Aspergillus fischeri* NRRL 181 XP_001259234.1, *Coniochaeta ligniaria* NRRL 30616 OIW35663, *Aureobasidium pullulans* AAR87862.1 and the two *Trichomonascus ciferrii* KAA8917114.1 and KAA8917113.1 conserved proteins.

Amino acids 21 to 701 of the *B. raffinosifermentans* Agu1 protein correspond to the COG3661 conserved domain of the alpha glucuronidase AGUA2 family.

Amino acids 705 to 839 of the *B. raffinosifermentans* Agu1 protein correspond to the Carbohydrate Binding Module 6 (CBM6) and CBM35_like superfamily (cd02795).

E in positions 705 and 707 and D in position 835 of the *B. raffinosifermentans* Agu1 protein are the three conserved residues that bind metal ion in the module (Nurizzo et al.; 2002).

N-glycosylation sites predicted in *B. raffinosifermentans* Agu1 protein, at positions 55, 156, 196, 286, 317, 349, 441, 533, 616, and 688.
