# Supplementary material for: Development of a Vector Set for High or Inducible Gene Expression and Protein Secretion in the Yeast Genus Blastobotrys
Source: J Fungi (Basel). 2022 Apr 19;8(5):418. doi: 10.3390/jof8050418 (PMC9144253; doi:10.3390/jof8050418)
Supplement: Supplementary file 1 [file jof-08-00418-s001.zip › FileS3.pdf]

File S3: Sequences and alignment of arabinoxylan alpha arabinofurano hydrolases (GH43 AXH-like)

```
>Apiotrichum.siamense_AXH arabinoxylan alpha arabinofuranohydrolase
MPSPLVSHIYTADPSAHVFDGRIYVYPSHDRETDIGFNDNGDQYDMVDYHVLSMDTIGGPVTDHGVALAAKDVPWVDKQLWAP
DAAKGADGRYYLYFPARAHDGIFRIGVAVSDKPEGPFTPQPEAIKSSFSIDPASFVDEDGTPYLYFGGLWGGQLQKYNADGTV
FTADAPEEPSGPGVRALGPRVARLRGDMLEFDETVKEILIVDDAGEPIPADDHERRFFEAAWMHKRNGVYYFSYSTGDTHLLA
YATGSSPYGPFTYQGTILEPVLGWTTHHSIVDFDNKTYLFYHDCELSKGVDHLRSVKCREIEYDAEGKIIQQKP

>Apiotrichum.porosum_XP_028476455.1
MSAPLVTHIYTADPSAHVFDGRVYVYPSHDRETDIHFNDNGDQYDMVDYHVLSMDKIGGEVTDHGVALAAKDVPWVDKQMWAP
DAAKGADGRYYLYFPARAHDGVFRIGVAVSDKPEGPFAPQPEAIKGSFSIDPASFVDEDGTAYLYFGGLWGGQLQRYNADGTV
SDPKAPEEPTGAGVRALGPRVARLRGDMLEFDETVREILIVDDEGNPILADDHERRFFEAAWMHKRNGKYYFSYSTGDTHLLA
YGIGTSPYGPFTYQGTILEPVVGWTTHHSIVDDFEGKSYLFYHDCELSKGVDHLRSVKCREIQYDAAGKIIKQTP

>Cutaneotrichosporon.oleaginosum_XP_018280189.1_xylosidase/arabinosidase
MPKPLVTHIYTADPSAHVFDGRLYIYPSHDRENDYGFNDKGDQYDMVDYHVLSLAEEGGEVTDHGVAISAQDVPWVDKQMWAP
DCARGKDGRHYLYFPARGHDGIFRIGVAVGDRPEGPFKAQPEPIKGSFSIDPATFVDDDGAAYCYFGGLWGGQLQCYNSDGTT
YTPDVADEPSGAGVRAYGPRVARLRDDMLEFAEGVRELVITVGGQPVPADDHERRFFEAAWVHKHNGRYYFSYSTGDTHLLAY
AIGDSPYGPFEYAGTILEPVVGWTTHHSIVENFKGKTYLFYHDCELSKGVDHLRSVKMREITYDKDGKIIKQKP

>Trichosporon.asahii var. asahii CBS2479_XP_014181348.1
MPEPLVKHIYTADPSAHAFAGKIYVYPSHDRETDIQFNDNGDQYDMVDYHVLSMDEVGGEVTDHGVALSAEQVPWVDKQMWAP
DCAEKNGKYYFYFPARAKDGKFKMGVAVSDKPEGPFTPQPEAMKGSFSIDPAAFVDDDGEAYLYFGGLWGGQLQCYNSDGTEW
DGSKQGPQEPTEGDALGPRVARLTDDMLEFAEPVREISITYQGKPVPASDHEKRFFEAAWMHKRNGKYYFSYSTGDTHLIAYA
TGDSPYGPFEYQGTVLTPPIGWTNHHSMVHFKGKDYLFYHDCSLSKGVDHLRSVKAKEMKFDGDKIVTMDP

>Humicola.insolens_AHC72382.1 Xyl43A
MAPLITNIYTADPSAHVFNGKLYIYPSHDRETDIQFNDNGDQYDMADYHVFSLDSLDPPSEVTDHGVVLKVEDIPWVSKQLWA
PDAATKDGKYYLYFPARDKEGIFRIGVAVSDKPEGPFTPDPEPIKGSYSIDPAVFVDDDGSAYMYFGGLWGGQLQCYQKGNNI
FDAEWSGPKEPSGSGAKALGPRVAKLTDDMRQFAEEVREIVILAPETGEPLAADDHDRRFFEAAWMHKYNGKYYFSYSTGDTH
YLVYAVGDSPYGPFTYGGRILEPVLGWTTHHSIVEFQGRWWLFHHDCELSKGVDHLRSVKVKEIWYDKDGKIVTEKPE

>Aspergillus.clavatus_NRRL 1_XP_001276579.1
MASEPLVKHIYTADPSAHVFNNKLYIYPSHDRETDIKFNDNGDQYDMNDYHVFSMETVGGPVTDHGVVLKDSDVPWVSKQLWA
PDAATKNGKYYLYFPARDQEGIFRIGVAVGDKPEGPFTAQPNPIRGSYSIDPATFVDDDGQAYLYFGGIWGGQLQCWSKSDAG
EWKFDASKTGPQEPAGEGVLALAPRVARLTDDMLEFDSEIKELLIVDPEGKPLAADDHERRFFEAAWMHKYNGKYYFSWSTGD
THYLVYATGDSPFGPFTYRGRILEPVLGWTTHHSIAEFQGKWYLFYHDCSLSNGVDHLRSVKMREIVYDAEGNIRLAEE

>Penicillium.roqueforti_XP_038925552.1
MPKPLVSHIYTADPSAHVFNNKLYIYPSHDRKTTITFNDNGDQYDMADYHVLSMDTITSPVTDHGVVLRDKDIPWVSKQLWAP
DAATKNNKFYLFFPARDHKGIFRIGVAVSQSPEGPFIPQDNYIQGTYSIDPAVFVDEEAGGEAYLYVGGIWGGQLQCWVEGKD
EDGGEGECVDGEGAGRKKRLVFDESRSGPQEPTGEGVRALCPRVALLRDDMLSLVSSMKEVVILAPETGEPLLADDHERRFFE
AAWVHRYQGTYYFSYSTGDTHFLAYATGDSPWGPFTYQGRILDPVLGWTTHHSIVEFQGKWYLFYHDCELSEGIDHLRSVKVR
EIVYDDEGAIHLAEEQTPV

>Aspergillus.fumigatus Af293_XP_747277.1
MSEPLVKHIYTADPSAHVFNNKLYIYPSHDRETDIQFNDNGDQYDMNDYHVFSLETVGGPVTDHGVVLKDSDIPWVSKQLWAP
DAATKNGKYYLYFPARDREGIFRIGVAVSDKPEGPFAPQPNPIRGSYSIDPAAFVDDDGQAYLYFGGIWGGQLQCWSRSAATG
EWEFDASKTGPQEPRGEGVKALYPRVARLTEDMLEFEGEIQELQIVDEERRPLAADDHERRFFEAAWMHKYNGKYYFSWSTGD
THYLVYATGDSPLGPFTYRGRILEPVLGWTTHHSIAEFQGKWYLFYHDCSLSGGVDHLRSVKMREIVYDAEGNIKLAE

>Fusarium.tjaetaba_XP_037205011.1
MAPLITNVYSADPSAHVFNGKIYVYPSHDRETDIKFNDNGDQYDMADYHVFSTESLDPPAEVVDHGVVLKTEDIPWVSKQLWA
PDAAEKNGKFYLYFPARDKQGIFRIGVAVGDKPEGPFTPDPEPIKGSYSIDPASFVDEDGQAYLYFGGLWGGQLQCYQKGNDI
FDPEWQGPKEVSGEGVPAQGPRVAKLTEDMHQFADEVKEIQILDPESGKPILGDDHDRRFFEAAWMHKRNGKYYFSYSTGDTH
FLCFATGDSPYGPFTYGGRILEPVLGWTTHHSIVEFKGKTYLFYHDCELSKGVDHLRSVKAKEIFYDDDGKIITTTAD

>Neurospora.crassa OR74A_EAA36295.3
MAPLITHMYTADPSAHVFNGRVYIYPSHDRETDIQFNDNGDQYDMADYHVFSTPSLDPQGPVTDHGVVLKTEDVPWVSKQLWA
PDAACKNGKYYLYFPARDKQGIFRIGVAVGDKPEGPFTPQPEPIKGSYSIDPATFVDDDGEAYLYFGGLWGGQLQCYQKGNNV
FDAEWQGPKEPTGKGVKSLGARVGRLTQNMLEFADEQGVREVTILAPETGEPLDADDHERRFFEAAWMHKRNGKYYFSYSTGD
THFLCYAVGDSPYGPFTYGGRILEPVLGWTTHHSIVEFPEKSDRWWLFHHDCELSKGVDHLRSVKVKEIFFDEEGKICGEKQP
AGK
```

```
>Colletotrichum.siamense_XP_036490221.1
MAPLIDHIYTADPSAHVFNGKIYIYPSHDRETDIQFNDNGDQYDMADYHVFSTSSLDPLEPVVDHGVVLKTEDVPWVSKQLWA
PDAAFKNGKYYLYFPARDKQDIFRIGVAVSDKPEGPFTPDPEPIKGSYSIDPASFVDDDGQAYLYFGGLWGGQLQCYQKGNDM
FDPEWLGPKEPSGKGVFALGPRVAKLTEDMHQFDGEVQEILILDPETKRPILGDDHERRFFEAAWMHKKDNLYYFSYSTGDSH
YLCYGTSESPFGPFTYGGRILEPVLGWTTHHSIVEFKGQWWLFHHDCELSNGVNHLRSVKVKEIFYDAEGRIITEKP
```

```
                       1       10         20        30        40        50        60        70        80        90        100       110       120      130
                       |--------+---------+---------+---------+---------+---------+---------+---------+---------+---------+---------+---------+---------|
Apiotrichum.siamense    MPSPLVSHIYTADPSAHVFDGRIYVYPSHDRETDIGFNDNGDQYDMVDYHVLSMDTIG--GPVTDHGVALAAKDVPWVDKQLWAPDAAKGADGRYYLYFPARAHDGIFRIGVAVSDKPEGPFTPQPEAI
Apiotrichum.porosum_    MSAPLVTHIYTADPSAHVFDGRVYVYPSHDRETDIHFNDNGDQYDMVDYHVLSMDKIG--GEVTDHGVALAAKDVPWVDKQMWAPDAAKGADGRYYLYFPARAHDGVFRIGVAVSDKPEGPFAPQPEAI
Cutaneotrichosporon.    MPKPLVTHIYTADPSAHVFDGRLYIYPSHDRENDYGFNDKGDQYDMVDYHVLSLAEEG--GEVTDHGVAISAQDVPWVDKQMWAPDCARGKDGRHYLYFPARGHDGIFRIGVAVGDRPEGPFKAQPEPI
 Trichosporon.asahii    MPEPLVKHIYTADPSAHAFAGKIYVYPSHDRETDIQFNDNGDQYDMVDYHVLSMDEVG--GEVTDHGVALSAEQVPWVDKQMWAPDCAE-KNGKYYFYFPARAKDGKFKMGVAVSDKPEGPFTPQPEAM
Humicola.insolens_AH     MAPLITNIYTADPSAHVFNGKLYIYPSHDRETDIQFNDNGDQYDMADYHVFSLDSLDPPSEVTDHGVVLKVEDIPWVSKQLWAPDAAT-KDGKYYLYFPARDKEGIFRIGVAVSDKPEGPFTPDPEPI
Fusarium.tjaetaba_XP     MAPLITNVYSADPSAHVFNGKIYVYPSHDRETDIKFNDNGDQYDMADYHVFSTESLDPPAEVVDHGVVLKTEDIPWVSKQLWAPDAAE-KNGKFYLYFPARDKQGIFRIGVAVGDKPEGPFTPDPEPI
Colletotrichum.siame     MAPLIDHIYTADPSAHVFNGKIYIYPSHDRETDIQFNDNGDQYDMADYHVFSTSSLDPLEPVVDHGVVLKTEDVPWVSKQLWAPDAAF-KNGKYYLYFPARDKQDIFRIGVAVSDKPEGPFTPDPEPI
   Neurospora.crassa     MAPLITHMYTADPSAHVFNGRVYIYPSHDRETDIQFNDNGDQYDMADYHVFSTPSLDPQGPVTDHGVVLKTEDVPWVSKQLWAPDAAC-KNGKYYLYFPARDKQGIFRIGVAVGDKPEGPFTPQPEPI
Aspergillus.clavatus   MASEPLVKHIYTADPSAHVFNNKLYIYPSHDRETDIKFNDNGDQYDMNDYHVFSMETVG--GPVTDHGVVLKDSDVPWVSKQLWAPDAAT-KNGKYYLYFPARDQEGIFRIGVAVGDKPEGPFTAQPNPI
Aspergillus.fumigatu    MSEPLVKHIYTADPSAHVFNNKLYIYPSHDRETDIQFNDNGDQYDMNDYHVFSLETVG--GPVTDHGVVLKDSDIPWVSKQLWAPDAAT-KNGKYYLYFPARDREGIFRIGVAVSDKPEGPFAPQPNPI
Penicillium.roquefor    MPKPLVSHIYTADPSAHVFNNKLYIYPSHDRKTTITFNDNGDQYDMADYHVLSMDTIT--SPVTDHGVVLRDKDIPWVSKQLWAPDAAT-KNNKFYLFFPARDHKGIFRIGVAVSQSPEGPFIPQDNYI

                       131     140        150       160       170       180       190       200       210       220       230       240       250      260
                       |--------+---------+---------+---------+---------+---------+---------+---------+---------+---------+---------+---------+---------|
Apiotrichum.siamense   KSSFSIDPASFVDED--GTPYLYFGGLWGGQLQKYNADGTV---------------------FTADAPEEPSGPGVRALGPRVARLRGDMLEFDET--VKEILIVDDA-GEPIPADDHERRFFEAAWMH
Apiotrichum.porosum_   KGSFSIDPASFVDED--GTAYLYFGGLWGGQLQRYNADGTV---------------------SDPKAPEEPTGAGVRALGPRVARLRGDMLEFDET--VREILIVDDE-GNPILADDHERRFFEAAWMH
Cutaneotrichosporon.   KGSFSIDPATFVDDD--GAAYCYFGGLWGGQLQCYNSDGTT---------------------YTPDVADEPSGAGVRAYGPRVARLRDDMLEFAEG--VRE-LVITVG-GQPVPADDHERRFFEAAWVH
 Trichosporon.asahii   KGSFSIDPAAFVDDD--GEAYLYFGGLWGGQLQCYNSDGTE---------------------WDGSKQGPQEPTEGDALGPRVARLTDDMLEFAEP--VRE-ISITYQ-GKPVPASDHEKRFFEAAWMH
Humicola.insolens_AH   KGSYSIDPAVFVDDD--GSAYMYFGGLWGGQLQCYQKGNNI-------------------FDAEWSGPKEPSGSGAKALGPRVAKLTDDMRQFAEE--VREIVILAPETGEPLAADDHDRRFFEAAWMH
Fusarium.tjaetaba_XP   KGSYSIDPASFVDED--GQAYLYFGGLWGGQLQCYQKGNDI-------------------FDPEWQGPKEVSGEGVPAQGPRVAKLTEDMHQFADE--VKEIQILDPESGKPILGDDHDRRFFEAAWMH
Colletotrichum.siame   KGSYSIDPASFVDDD--GQAYLYFGGLWGGQLQCYQKGNDM-------------------FDPEWLGPKEPSGKGVFALGPRVAKLTEDMHQFDGE--VQEILILDPETKRPILGDDHERRFFEAAWMH
   Neurospora.crassa   KGSYSIDPATFVDDD--GEAYLYFGGLWGGQLQCYQKGNNV-------------------FDAEWQGPKEPTGKGVKSLGARVGRLTQNMLEFADEQGVREVTILAPETGEPLDADDHERRFFEAAWMH
Aspergillus.clavatus   RGSYSIDPATFVDDD--GQAYLYFGGIWGGQLQCW--SKSDA-GEWK-------------FDASKTGPQEPAGEGVLALAPRVARLTDDMLEFDSE--IKELLIVDPE-GKPLAADDHERRFFEAAWMH
Aspergillus.fumigatu   RGSYSIDPAAFVDDD--GQAYLYFGGIWGGQLQCW--SRSAATGEWE-------------FDASKTGPQEPRGEGVKALYPRVARLTEDMLEFEGE--IQELQIVDEE-RRPLAADDHERRFFEAAWMH
Penicillium.roquefor   QGTYSIDPAVFVDEEAGGEAYLYVGGIWGGQLQCWVEGKDEDGGEGECVDGEGAGRKKRLVFDESRSGPQEPTGEGVRALCPRVALLRDDMLSLVSS--MKEVVILAPETGEPLLADDHERRFFEAAWVH

                       261     270        280       290       300       310       320       330       340       350       360
                       |--------+---------+---------+---------+---------+---------+---------+---------+---------+---------|
Apiotrichum.siamense   KRNGVYYFSYSTGDTHLLAYATGSSPYGPFTYQGTILEPVLGWTTHHSIVD---FDNKTYLFYHDCELSKGVDHLRSVKCREIEYDAEGKIIQQKP
Apiotrichum.porosum_   KRNGKYYFSYSTGDTHLLAYGIGTSPYGPFTYQGTILEPVVGWTTHHSIVDD--FEGKSYLFYHDCELSKGVDHLRSVKCREIQYDAAGKIIKQTP
Cutaneotrichosporon.   KHNGRYYFSYSTGDTHLLAYAIGDSPYGPFEYAGTILEPVVGWTTHHSIVEN--FKGKTYLFYHDCELSKGVDHLRSVKMREITYDKDGKIIKQKP
 Trichosporon.asahii   KRNGKYYFSYSTGDTHLIAYATGDSPYGPFEYQGTVLTPPIGWTNHHSMVH---FKGKDYLFYHDCSLSKGVDHLRSVKAKEMKFDGD-KIVTMDP
Humicola.insolens_AH   KYNGKYYFSYSTGDTHYLVYAVGDSPYGPFTYGGRILEPVLGWTTHHSIVEF---QGRWWLFHHDCELSKGVDHLRSVKVKEIWYDKDGKIVTEKPE
Fusarium.tjaetaba_XP   KRNGKYYFSYSTGDTHFLCFATGDSPYGPFTYGGRILEPVLGWTTHHSIVEF---KGKTYLFYHDCELSKGVDHLRSVKAKEIFYDDDGKIITTTAD
Colletotrichum.siame   KKDNLYYFSYSTGDSHYLCYGTSESPFGPFTYGGRILEPVLGWTTHHSIVEF---KGQWWLFHHDCELSNGVNHLRSVKVKEIFYDAEGRIITEKP
   Neurospora.crassa   KRNGKYYFSYSTGDTHFLCYAVGDSPYGPFTYGGRILEPVLGWTTHHSIVEFPEKSDRWWLFHHDCELSKGVDHLRSVKVKEIFFDEEGKICGEKQPAGK
Aspergillus.clavatus   KYNGKYYFSWSTGDTHYLVYATGDSPFGPFTYRGRILEPVLGWTTHHSIAEF---QGKWYLFYHDCSLSNGVDHLRSVKMREIVYDAEGNIRLAEE
Aspergillus.fumigatu   KYNGKYYFSWSTGDTHYLVYATGDSPLGPFTYRGRILEPVLGWTTHHSIAEF---QGKWYLFYHDCSLSGGVDHLRSVKMREIVYDAEGNIKLAE
Penicillium.roquefor   RYQGTYYFSYSTGDTHFLAYATGDSPWGPFTYQGRILDPVLGWTTHHSIVEF---QGKWYLFYHDCELSEGIDHLRSVKVREIVYDDEGAIHLAEEQTPV
```

Alignments were performed with the Multalin server (http://multalin.toulouse.inra.fr/multalin/). The *Apiotrichum siamense* Axh1 predicted protein was aligned with Axh1 proteins from Basidiomycetes: *Apiotrichum porosum* XP_028476455.1, *Cutaneotrichosporon oleaginosum* XP_018280189.1 and *Trichosporon asahii var. asahii* CBS2479 XP_014181348.1 and Axh1 proteins from Ascomycetes: *Humicola insolens* AHC72382.1 Xyl43A, *Aspergillus clavatus* NRRL 1 XP_001276579.1, *Penicillium roqueforti* XP_038925552.1, *Aspergillus fumigatus* Af293 XP_747277.1, *Fusarium tjaetaba* XP_037205011.1, *Neurospora crassa* OR74A EAA36295.3 and *Colletotrichum siamense* XP_036490221.1.

⬇ Acidic amino acids that form the active sites as defined for the conserved domain cd08990 (Pons et al., 2004): D in position 13, D in position 134 and E in position 225 for the *A. siamense* Axh1 protein.
