# Supplementary material for: Development of a Vector Set for High or Inducible Gene Expression and Protein Secretion in the Yeast Genus Blastobotrys
Source: J Fungi (Basel). 2022 Apr 19;8(5):418. doi: 10.3390/jof8050418 (PMC9144253; doi:10.3390/jof8050418)
Supplement: Supplementary file 1 [file jof-08-00418-s001.zip › FileS4.pdf]

File S4: Sequences and alignment of beta-xylosidase (GH3)

```
>1_Blastobotrys.raffinosifermentans_ARAD1D50644
MIFSLLPFAGLALAQAQTGYVNYSQEAQPNLTPQTVETITIKYPDCSSGPLANTVVCNASADYRERAWALVSMMTFDELVNNTQNTAPGVPRLG
LPPYQWWNEALHGLDRANFSAEVSEEFGWATSFPQPILTAAALNRSLIHTIGDIISTQARAFSNNGKYGLDAYSPNINGFRSPLWGRGQETPGE
DANFLSSVYAYEYITGLQGPDAPNQIKIAATAKHFAGYDLENWDGHSRLGNDLFISQQDLAEYYTPQFLTAIRDAKSVSLMCSYNAVNGVPSCA
SPYFLQTILRESWGFDRYDGYVSSDCDAVYNVFNPHNYSSTDALAAADSMRAGTDLDCGQEYPWHLTEAFANGDITRSEVELAVYRLYATLVRL
GYFDTNHPYRKLGWKDVVDTNNLNVTYEAAVQGITLLKNDGTLPLKEGKSVALIGPYANATEQMQGNYFGPAPYVITPLQAAKSMGLKATYAFG
TNISSTSKEFFDDALKVAKESDEIIFFGGIDNTIEAEGMDRMNLTWPGNQLELISELSKCGKPLTVVQFGGGQVDSSSLKNNHNVNSLVWAGYP
GQSAGAAIMDILTGKRAPAGRLVTTQYPAEYAKQLPATDMSLRPNKSTNNPGQTYMWYTGKPVYEFGHGLFYTTFKTDLTSEAKNTFNLTKVLT
AAHPGYDSPDLQTLLNVTVTVTNTGDVASDYTNMLFANTKNAGPAPYPNKWLVGFDRLSSIQPGSSKTLTIPVPVGALSRVDEDGNRVLYPGDY
ELHLGTDKEDKVKASVKLTGKAVTLTHWPADNF

>2_Blastobotrys.raffinosifermentans_ARAD1C05676
MKWTTSALTAVAVSGVQAANWFGPPYPDQNEFPDCANGPLKDNDVCNTELDPITRAKGLVSAMTREEKIVNVQHNATGVPRLGLAPYNWWSEAL
HGVASSPAVVFEDSGDFSSATSFPQPILMGAAFDDKLIFDVATVVGTEARAFGNHQKAGLDFWTPNINPYRDPRWGRGQETPGEDPYHLQNYVY
NLLSGLEGPKDEPYKRIVATCKHYAGYDLENWNGIERHEFDAKISTQDLSEFYTPSFRTCARDAKVGAFMCTYNMVNGMPTCGNPYFLDTLLRD
HWNWRGIGNWVTGDCGAVEDIYKTHNYTKDAASAAALALNAGTDLDCGETFPEGLPAAFDQGQVKESTLDQALVRLYSSLVHLGYFDPEGSQKY
RRLGWSDVNQPHAQDLAYKAAVEGIVLLKNDGILPIKPDGQKVAVVGPLSQATTQMQGNYFGVAPHLHSPQYGAQQQNWQVSQANGTFIESTNR
TGFDDAISAAKDADVIFYVGGIDNSIELEGLDRMTISWPQNQLDLIDELSKLGKKLVVVQMGGGQVDDTPLKNNDKINAIVWAGYPGQDGGVAI
FDIITGKVSPAGRLPVTQYPAEYVDQVPMTDMDLRPSDSNPGRTYRWYDKAVYEFGYGLHYTDFEVSFKGNKDKKYKVDQVIHSGKKVDNLDQK
VFDTFKVEVENKGDVTSDYIALAFIKTDNAGPGPYPLKTLAGYTRAFSIAPGEKRKVDIDVPVGQVARVDEKGNRVLYPGDYTLEIDVDGANGG
ASISFSISGDPYTIEEFPQPDF

>1_Blastobotrys.adeninivorans_XYL1
MIFSLLPFAGLALAQAQTGYVDYNREAQPNLTPQSAGTITVKYPDCSNGPLANTIVCNASADYRERAWALVSMLSFDELVNNTQNTSPGVPRLG
LPPYQWWNEALHGLDRANFSADVGEEFGWATSFPQPILTAAALNRTLIHTIADIISTQARAFSNNGKYGLDAYSPNINGFRSPMWGRGQETPGE
DANFLSSVYAYEYITGLQGPDAPNNIKIAATAKHFAGYDLENWDGHSRLGNDLTISQQDLAEYYTPQFLTAIRDAKSVSLMCSYNAVNGVPSCA
NPYFLQTILRESWGFDRYDGYVSSDCDAVYNVFNPHNYSSTDALAAADSMRAGTDLDCGQEYPWHLTEAFANGDITRSEVELAVYRLFATLVRL
GYFDTNHPYRQLGWKDVVDTNNLNVTYEAAVQGITLLKNDGTLPLKQGKSVALIGPYANATEQMQGNYFGPAPYIITPLQAAKSMGLKATYAFG
TNISSTSKEFFDDALKVAKESDEIIFFGGIDNTVEAEGVDRMNVTWPGNQLELISELSKCGKPLTVVQFGGGQVDSSSLKDNHNVNSIVWAGYP
GQSAGAAIMDILTGKRAPAGRLVTTQYPAEYANQFPATDMSLRPNKSTNNPGQTYMWYTGKPVYEFGHGLFYTTFKIDLTSEAKNTFNLTEVLS
TPHPGYDSPDLQTLLNVTVTVTNTGDVASDYTNMLFANTKNAGPAPYPNKWLVGFDRLSSIQPGSSQTLTIPVPVGALSRVDEDGNRVLYPGDY
ELNLGTDNEDKVKASVKLTGKAVTLTHWPADDNF

>2_Blastobotrys.adeninivorans_XYL1
MKWTTSALTAIAVSGVQAAEWFGPHYPNQNEFPDCGNGPLKDNDICNTDLDPITRAKGLVSAMTRDEKIVNVQHNATGVPRLGLAPYNWWSEAL
HGVAESPAVVFEESGDFSSATSFPQPILMGAAFDDKLIFDIATVVGTEARAFGNNQKAGLDFWTPNINPYRDPRWGRGQETPGEDPYHLQNYVY
SLLSGLEGPKDEPYKRIVATCKHYAGYDLENWNGITRHEFDAQISTQDLSEFYTPSFRTCARDAKVGAFMCTYNMVNGIPTCGNPYFLDTLLRD
HWNWRGIGNWVTGDCGAVEDIYKYHNYTKDAASAAALALNAGTDLDCGETYPEGLPAAFDQDQVKESTLDQALVRLYSSLVHLGYFDPEGSQKY
RRLGWSDVNQPHAQELAYKAAVEGIVLLKNDGFLPVKPEGQKVAVVGPWSQATGQMQGNYLGVAPFLHSPLYGAQQQDWQVSQANGTFIQSTNR
TGFDDAISAAKDADVIFYVGGIDNTIELEGLDRMNISWPQNQLDLIDELTKLGKKLVVVQMGGGQLDDTPLKNNDKINAILWGGYPGQDGGVAI
FDIITGKAAPAGRLPVTQYPAEYADQVPMTDMSLRPSDSNPGRTYRWYDKAVYQFGYGLHYTDFEVSFKGNKDKKYKVDQVIHSGKKVENLDQK
VFDTFKVEVENKGDVTSDYVALAFIKTDNAGPAPYPLKTLAGYTRAFSIGPGEKRKVDIDVPIGQVARVDEKGNRVLYPGDYTLEIDVDGANGG
ASVSFSVSGDPFTIEEFPQPDF

>Blastobotrys.mokoenaii_XYL1
MVSSGTLLTAVATALLATPALAQNNQTYANYSEKAQPDLYPLTLANIKRGFPDCENGPLKDNLVCDTSANYFERAQALISLFTTEELVNNTRNS
GPGVPRLGLPPYQVWNEALHGLARANETKEGEWSWSTSFPQPILSMAAFNRTLINQIGDIIATQARAFSNEERFGLNVYSPNINGFRSPLWGRG
QETPGEDAFFLSSVYAYEYITGLQGGVDPENLKIAATAKHFAGYDLENWGNVSRLGLNAEITQQELSEYYTPQFLAASRYAKTYSLMCSYNSVN
GVPSCANSFLLQTLLRDSWDFPEHGYVSSDCGAVYNVFNPHGYAHNTTSAAADALRAGTDIDCGETFPDFLDDSFDEGYVSRGEIEKGLTRLYA
TLVRLGYFDGKNAKYRDLSWNDVVQTDSWNISYEAAVEGITLLKNDGTLPLSKSVKKVALIGPWANATEQLLGNYFGTPPYMVSPLQGAKDAGY
EVNFATGTGISDGSTEGYSDAIAAAKKSDVIIFAGGIDNTIEAEGQDRTDIAWPGKQLDLIDKLSKLGKPLVVLQMGGGQVDSSSLKKNKNVNS
LVWGGYPGQSGGHAIFDILRGKRAPAGRLVTTQYPAEYAHLFPAYDMNLRPNGSNPGQTYIWYTGEPVYEFGHGLFYTKFKETRSKGIKEHQTF
NLQDLLEKPHPGYKYPELAPFLNFTVDVKNTGHTESPYTGLVYARTTAGPKPLPNKWLVGFDRLPEIKPGHSSTMTIPIPIGAIVRADENGSKT
LYPGDYELALNNERSVVITFTLTGEPVTMENWPLYHQAVDGYVQTTIAGPVH

>Sugiyamaella.lignohabitans_XP_018735137.1 hypothetical protein AWJ20_922
MKSLTQLSAAIGLVGSALAQNQGNNYVNFSSEANPQLTSYTLEHLNYSFPDCTREPLKSNLVCDTSAHYLDRARALVNEFTLQELINNTDNTAP
GVPRLGIPNYQWWSEALHGIASSPGMQYAESGEYSHSTSFPQPILMGAAFDDDLIKRVATVVSTEGRAFNNKGLYGLDVWSPNINPFKDPRWGR
GQETPGEDPFHISGYVYNLIQGLQGGLNPDQLKLVATCKHFAGYDLESWEEHSRLGFDAIISERDLVEYYLPAFQSCVRDAKAASVMCSYNAVN
GVPSCANDFFLSSILRKEWGFDEYNGYVTSDCDAVYNVWNPHNYSDTEFGAAAASINAGCDLNCGQTYSDTLPQAFAQNLVTRDTIEDSVVRLY
ANLVRLGFFDPANTQPYRQLGWTDVSTNSSNQLAYQAAVEGIALLKNDGTLPLNSQIKKLALIGPWANATTQMQGNYEGVAPFLISPLQAAQNA
GFQVQYNIGTLINSTSTANFSQALQIAKDADAVIYVGGIDNTIEAEAQDRVNITWPGNQLDLISQLSGLSKPLIVLQMGGGQIDSSSLKNNSNV
NGLLWGGYPGQSGGQAILDILTGVVAPAGRLPTTQYPADYINQVPMTNMSLRAGDNNVGRTYMWYTGEPVYEFGTGLHYTTFDASWVNNNTATF
NIQDLIQNAHPGVDYVDQINFATFTAGITNTGKVASDYVALLFASGDAGPAPQPKKRLVSYSRAHDIAPGQTAQVKLPLTLGAISRVDENGNRV
LYPGTYNLALDTTGLITTTVTLTGNAQIITAWPTLGDSDFVKGQLLTQL

>Trichomonascus.ciferrii_KAA8906613.1
MRVSTLSYWTIASSLASLVSTAYPDCSKEPLKSNDVCNTELDAKQRAAALIKEWTLEEKINNTQNASPGVSRLGIPPYDWWNEALHGVADRHGV
FFAEPGEEFSYASSFPSPITMGSAFDDDLIFEVASTIGKEARAFGNGNHSGFDYWTPNINPFKDPRWGRGQETPGEDPIHLQKYVYKLLSGLEG
DDDEYLQIAATCKHYAAYDLEDWQGMDRFHFDAKVSMQDLNEYYLPTFRTCGRDFKVGAFMCSYNSINGEPGCANSYLLQDVLRDHWGWDRDDQ
WVTSDCGAIENIWHDHNFTKYDYQASAKALLAGTDLDCGTTFNNSLQEAYDEKLIKESDLDKALVRLYTSLVRTGYFDPEDKVSYRQLGWKDVN
TEEAQKLTYEAAVEGIVLLDNKKDTLPLSADKCGKVAVVGPYANATDLMLGNYHGIPPYHISPLDAVKKMGLDVSFYDQIPMNSTNSTGIDEAL
EAAKNADTILYFGGIDQTIEQEGHDRDTIVWPENQLKLIDKLAELKKTLVVIQFGGGQVDDTPLLDNDNVNAIVWTGYPGQEGGHAILDILTGK
KAPAARLPITQYPGDYVDQVPMTDMSLRPSDSNPGRTYRWYNEEVLPFGYGLHYTTFDVSWADEPADSYDITDVVQSAKQNAEYVDKAPFDTYK
```

VSVKNTGDVASDYVALLFLKTQNAGPAPYPRKTLIGYARAFDVQPGESQTVSIEVEVGAIARYDEQGNAVLYNGDYALQVDLEDNKGPTAKFSL
SGKSERIEEFPQPK

>1_Spathaspora.passalidarum_NRRL Y-27907_XP_007373774.1
MLPIKLLATLALAIFANGETPPSSPDYNTEANPQLPPITFEAVHYTFPDCQNGPLKHNAVCNPHLPTEQRAKAVVDLFTVDELIANMGNTSPGV
ERLGLPPYQWWSEALHGIARSNFTASGEYSHATSFPQPILMGGAFNNDLYKQVGNVIGTEARAFNNVGRAGLDFYSPNINPFRDARWGRGQEVA
SESPVLVGNYALNYVQGLQGGLDSNQNDDTLQVAATCKHFVGYDMESWNQHSRLGYNAIISDQDLADFYLPTFQSCVRDAKAAGAMCSYNAVNG
VPACASEFFLNTVLRDGFDFQNGVIHSDCDAIYNVWNPHLYAQDLGGAAADAIKAGVDVNCGDTYQNNLGYALGNKTINENQIRTSVTRQYSNL
IRLGYFDSPQTNKYRKYDWNDVSTPQANQLAYQAAVEGIALLKNDGTLPFNKQKVRKVAVIGPWANATTQMLGDYAGTPPYMISPLQGAQSEGF
QVEYALGTQINTTDTSGYTAALNAAKGADAIVYFGGIDNSVENEALDRESLAWPGNQLDLVSKLSGLKKPLVVLQFGGGQIDDTEIKNNKNVNA
IVYAGYPGQSGGTAIWDILSGKYAPAGRLTTTQYPASYADQVPMTDMTLRPRQGYPGRTFMWYNGEPVYEFGYGLHYTTFSASLANAPRGGHQS
FNIEQVVAAAKRSQYVDTGLITTFDVNIKNTGKTTSDYAALLYSKTTAGPGPHPNKILVSFDKLHQIHAGQTQTAKLPVTIGSLLQTDTNGNKW
LYPGTYTFFVDNDKKAQWEITLTGQAELIQKYPSQK

>2_Spathaspora.passalidarum_NRRL Y-27907_XP_007375862.1
MLPIKLLATLALSIFAIGETPPSFPDYNTESNPQLPPITFQAVHFSFPDCQNGPLKDNDVCNPYLPNNQRAKAVVDLFTVDELIANMGNTSPGV
ERLGLPPYQWWSEGLHGIARSNFTASGEYSHATSFPQPILMGGAFNSDLYKQVGNVIGTEARAFNNVGRAGLDYYSPNINPFKDPRWGRGQEVA
SESPVLVGNYALNYVQGLQGGIDSNPNDDTLQVAATCKHFAGYDMESWKQHSRLGYNAIISDQDLADYYFPTFQSCVRDAKAAGAMCSYNAING
IPVCASEFFLGTVIREGFDFQNGVIHSDCDSLYSIWNPHLYVQDLGAAAADGIKAGVDVNCGDTYQNNLGYALGNKTINEDQIRASVTRQYSNL
IRLGYFDSPQTNKYRTYNWSDVSTSQANQLAYQAAVEGITLLKNDGTLPFNKDKVKNVAVIGPWANATTDMLGDYAGTPPYLISPLQGAQDSGF
KVQYAYGTQINTTLTTNYTAALNAAKGADAIVYFGGIDNSIENEALDRESLAWPGNQLDLVSKLSGLNKPLVVVQFGAGQVDDTEIKNNNNVNS
IVYAGYPGQSGGTAIWDVLNGIYAPAGRLSTTQYPASYADQVPMTDMTLRPRDGYPGRTFMWYNGEPVYEFGYGLHYTTFSVSLANAPPKGAPQ
SFNIDQFIAAKSSQYVDTSLITTFDVNIKNTGKVTSDYAALLYSNTTSGPGPHPNKILVSFDKLHQIHPGQIQTASLPVTIGSLLQTDTNGNKW
LYPGAYTFFVDNDMKAQWEITLTGQAALVQNYPSQS

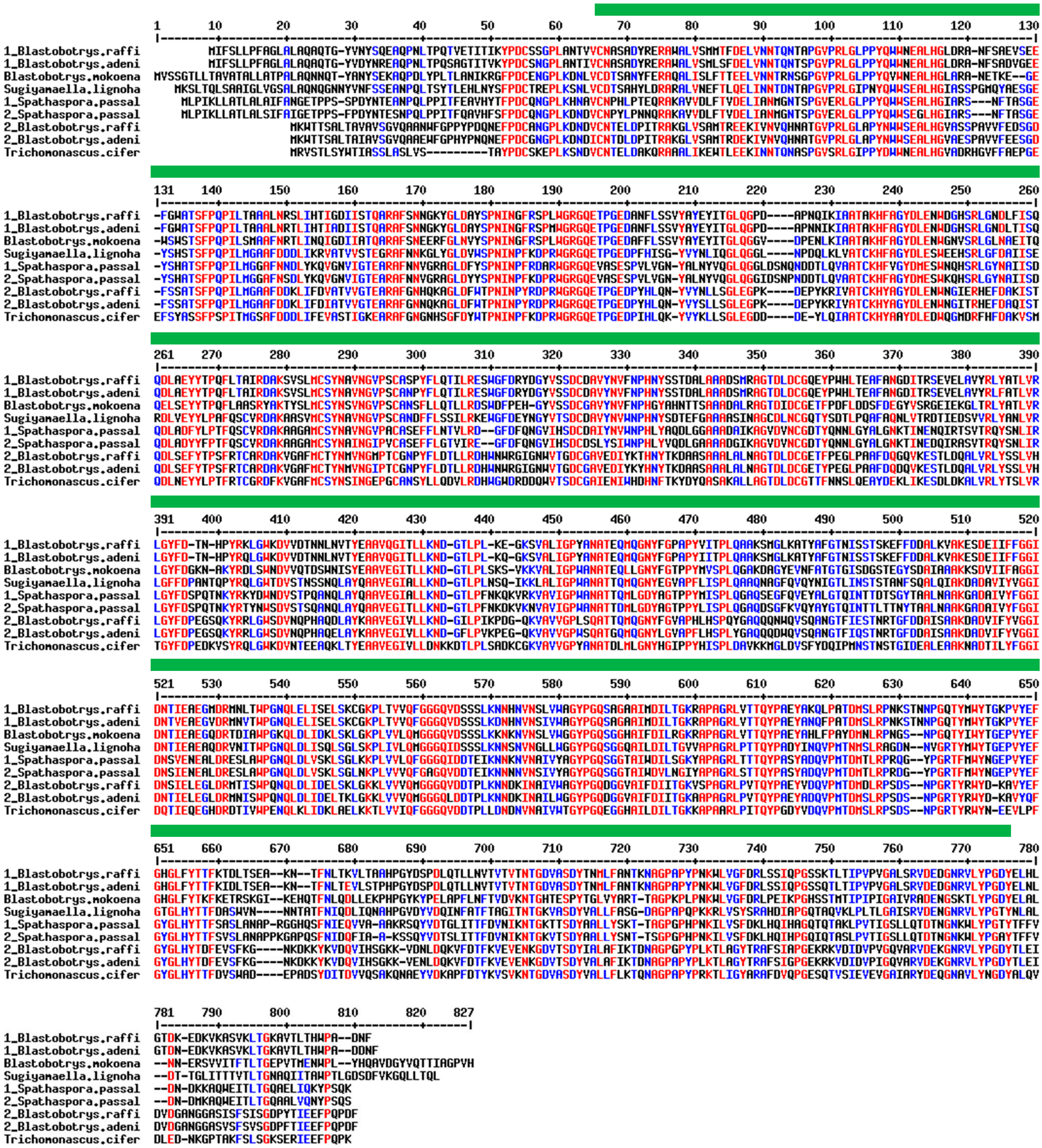

Alignments were performed with the Multalin server (http://multalin.toulouse.inra.fr/multalin/). The two *B. raffinosifermentans* ARAD1D50644 and ARAD1C05676 predicted proteins were aligned with two *B. adeninivorans* Xyl1 (personal data), *Blastobotrys mokoenaii* Xyl1, *Sugiyamaella lignohabitans* XP_018735137.1 *Trichomonascus ciferrii* KAA8906613.1 and *Spathaspora passalidarum* XP_007373774.1 and XP_007375862.1 conserved proteins

Amino acids 57 to 752 of the *B. raffinosifermentans* ARAD1D50644 protein and 46 to 740 of the *B. raffinosifermentans* ARAD1C05676 protein correspond to the conserved PLN03080 beta-xylosidase domain
