# Supplementary material for: Development of a Vector Set for High or Inducible Gene Expression and Protein Secretion in the Yeast Genus Blastobotrys
Source: J Fungi (Basel). 2022 Apr 19;8(5):418. doi: 10.3390/jof8050418 (PMC9144253; doi:10.3390/jof8050418)
Supplement: Supplementary file 1 [file jof-08-00418-s001.zip › FileS5.pdf]

File S5: Sequences and alignment of feruloyl esterase

```
>1_Blastobotrys.raffinosifermetans_ARAD1A06094
MASIPFFVEMKHFLGQSLLTSLLAAGAFGSSLAEVCTSSRIRTALPKDGAIAGISMDPDSITANPVYNASAGYSVFYPEGNFDYCNVTVSYCHI
GKGDKVNLQYWLPSPDKFQNRYLATGGGGYAINSGTQSLPGGVMYGAVAGRTDGGFGGFDVQVSEAILYANGSLNYDSLYMFGYRAIGEQTMIG
QELARGFYELGDEKKIYTYYQGCSEGGREGWSQIQKFPDLYDGVIPAAPAFRYGHQQVNHLFPGVIEQGMNYYPPPCEMARIVNATIEACDKLD
GKIDGVVSRTDLCLLNFDFNSTIGLHYTCEAGSNPMTGDSTPAQNGTVSTKAAELARVLTEGLHDSQGNKAYVFYQITAGYDDADTKYNPATGQ
FELSVSSLGGEWVTKFLQLVDLDNLPNLDNVTVDTLVDWMQCGWQTYEDVLQTTRPDLSLYERAGGKILTFHGESDNSIPAGSSVHFYESVRNV
MYPGISFNQSTDAMGEWYRLYLVPGAAHCSINALQPNGPFPQTTLEVMIDWVENGNTPTTLQATYLVGDNKGKPAEICPWPLRPTWTDEGSKLQ
CVYDHTSINTWMYDFNAFSLPVY

>2_Blastobotrys.raffinosifermetans_ARAD1A19822
MKNVILPIIATLLASTAYATASLADVCTSSYVRQAMPSNGTIEGITVNLSSVTANPVYNASSSGQVFFPDATYDYCNVTVSYSHNGLQDNVNLY
FWLPSPSAFQNRYLATGGGGFAINSGTGSLPGGVMYGAVAGSTDGGFGSFNNELTDVILYANGTLNYDALYMFGYRAIGEQTMIGKELAKNFYK
TDDKIYTYYQGCSEGGRDGWSQVQKYPELYDGVIPGAPAFRYGQQQVNHLTPNVMEQTMDYYPPPCELSKIVNATIEACDKLDGLEDGVVSRTD
LCMLHFNVNSTIGMPYYCAASSGQGGPMGGMGGSTSSTPAQNGTVSAKGAALVQTMLDGLHDLQGRRAYISYRISAEFSDAQTEYNSDTDSFEL
SINSQSGEWVTKFLQLVDTDNLSTLDGVTYDTLRDWMELGWQRYEDVLQTTWPDLSKYKEKGGKILHFHGESDPSIPAGSSVHYYDSVRSIMFP
DQSYNASIEAMNDFYRLYLIPGAAHCSTNQLQPNGPFPQTNLAVMIDWVEKGIAPNRLNATHLGGDYKGKSAEICPWPLRPLWTNNGTSLNCVF
DRTSLNTWKYKFDAYKLPLY*

>Aspergillus.niger_CBS 101883_XP_025455234.1 tannase and feruloyl esterase
MAGLSATAAHAASLADVCTTSNVKAALPSSVYGLTMIPSSVTASPVYNASTSGQVFFPDATYDYCGVTFNYTHNGRGDTVKLQYWLPAPESFEN
RFLATGGMAYMITGGSSYLPGGVMYGAVAGTTDGGFGGELDTAFLLANGTINYEALYSMGYHGIGELTIVGKEFTKNFYSMGDEKLYTYYQGCS
EGGREGWSQVQKYPGVYDGVIPGAPAIRYGQQQANHLYSNVVEKTLGYYPPPCELEKIVNATIDACDSLDGKVDGVVARTDLCQLHFNINSTIG
LPYHCAASSSSSIGLNYGKRSTDPAINGTVSAQGVAVAAEILKGLHDSKGRRAYISYQPTASFDDAETSYNSETGEYELSIASSGGEWVAKFLE
LRDADNLSTLDNVTYDTLRDWMELGWKRYEDVMQTTWPDLTEFEKAGGKIITFHGESDQSIHTGSSVHFYNSVRSIMYPDMSYNASVAAMGDWY
RLFLVPGAAHCATNDLQANGPFPQTNLAVMINWVEKGVVPHTLNATHLAGEWEGQNAQLCSWPLRPLWTENGTKFNCVYDQASVDTWDYNFDSY
KVPLY

>Aspergillus.heteromorphus_CBS 117.55_XP_025395144.1 tannase and feruloyl esterase
MRRAALTAGLSATTAYAASLADVCTTSNVKAALPASGAIQGLTMIPSSVTASPVYNASSSGEVFFPDATYDYCGVTFNYTHNGRDDTVALTYWL
PAPESFENRFLATGGMAYMISAGSAYLPGGVMYGAVAGTTDGGFGGSLDTVFLLANGTINYEALYSMGYHGIGEMTMVGKEFTKNFYNMTDDNK
LYTYYQGCSEGGRAGWSQVQKYPQVYDGIIPGAPAIRYGQQQANHLYSNVVEQTLGYYPPSCELERIVNATIDACDSLDGKVDGVVARTDLCML
NFDLNSTIGLSYSCAASSSSGIGLGFGKRSDPASNGTVSAEGVAVASEILKGLHDSQGRRAYISYQPSASFSDAATEYNSATGEYELDIASTGG
EWIVRFLELIDADNLSSLTNVTYDVLRDWMELGWKRYEDVLQTTWPDLSEFEQAGGKILTFHGESDPSIPTGSSVHFYESVRSIMYPELGYNAS
VDAMGDWYRLYMVPGAAHCATNDVQANGPFPQTNLAVMIDWVENGVTPTTLNATHLAGEWEGDNAQLCAWPLRPLWTENGTQFNCVYDQPSIDT
WNYTFDSYKVPLY

>Aspergillus.ellipticus_CBS 707.79_PYH90982.1 tannase and feruloyl esterase
MRRAALTAGLGATTAYAASLADVCTTANVKAALPASGAIDGLTMIPSSVSASPVYNASTSGQVFFPDATYDYCDVTFNYTHNGRGDTVALEYWL
PSPDTFENRFLATGGMAYMISGGTTSLPGGVQYGAVAGTTDGGFGGSLDTVFLLANGTINYEALYSMGYHGIGELTLVGKEFTKNFYSMGDDKL
YTYYQGCSEGGREGWSQVQKYPGVYDGVIPGAPAIRYGQQQANHLYSNVVEQTLGYYPPPCELERIVNATIDACDSLDGKVDGVVARTDLCMLH
FDINSTIGLSYSCAASSGGGIGLGFGKRSDPASNGTVSAQGVAVASEILKGLHDSQGRRAYISYQPSASFTDAETEYNSATGEYELDIASTGGE
WIAKFLELRDADNLSTLENVTYDVLRDWMELGWKRYEDVMQTTWPDLTEFEAAGGKIITFHGESDPSIPTGSSVHFYESVRSIMYPELAYNASV
SAMGDWYRLFLVPGAAHCATNDVQANGPFPQTNLAVMINWVEKGITPTTLNATHLAGDYEGDNAQLCAWPLRPYWTENGTQFNCVYDQPSIDTW
NYTFDSYKLPLY

>Ophiobolus.disseminans_KAF2833293.1 feruloyl esteras-like protein B precursor
MHFYQSLAAGAAYFTIIHAVSLADTCTVSRVLASLPANGTFLGISLDSTSITANAVYNASNTAQPFFPAATYDYCNVTLSYSHNARGDVVNLQY
WFPTPDNFKNRYLSTGGGGYAINSGSQGFPGGVMYGAVAGSTDGGFGSFKTKLDAVVLKANGTLNWDAIFMFGYQAIGELTQIGKGITRNFYSI
GEDDKLYTYYQACSEGGREGMSQIQRYGEEYDGVIPGAPALRYAQQQVQHLYSNVVEKTLDYFPSSCEFQRIVNATIKFCDPLDGKTDGVVART
DLCKLQFNINSTIGLPYSCAASSAAGLGFNFSSKSKRQMMPGGGASTPAANGTVSAQAVAVASTIMSGLHTSDGQFAYFSYQPSSSFEDAKTTY
NNETDSYELSIASTGGEFVTKFIQMVDADNLASLDNVTYDTLVEWMREAWIKYEDTLQTTLPDLTPFYNNGGKVLTFHGESDNSIPAASSVHYH
ESVRSIMYPNSTFNASTEAMGEWYRLFLVPGAGHCGVNSMQPNGPFPTTNLEVLIEWVENGVKPETLRATHTAGDEKGQSAEICAWPLRPYWVN
NGTNMECVYDQASVDSWMFEFDAWKMPLY

>Lophiotrema.nucula_KAF2119681.1 Tannase/feruloyl esterase
MKPQRAFLGSVFTTVVHSTALDELCTPSHVQASLPANGSISGIAPIPSSVTANAVYNASVSVSDNYPAATFDYCNVTFSYTHVGRNDIVNLQYW
LPSPDKFQKRYLATGGGGFAINSGTMMLPSGLQNGAVAGVTDGGFGSFSTQLDAIFLLANGTINYEPLFMFGYRAIGEQTKVGKALAKNFFSMN
STEKLYTYYQGCSEGGREGWSQVQRYPEEYDGVIPGAPAFRFAQQQVQHLYSNVVEQTLDYYPSTCEFDKIANETIKACDSLDGKTDGVVARTD
LCILQFNVSSLVGTPYFCTASAGSSPFPGGPAGSPTPVQNGTISAKGAEVAQTILNGLHDSQGRRAYISYQPATTFVDGATKYNNITGEWELSI
NSMGGEYVTKFLQLVNISNLPNLDNVTYDTLRDWMQQGWTMYEDTLQTTLPDLTPYQSKGGKVLHWHGESDNSIPTGSSVHYHESVRSVMYPSL
SFNESSAALSDWYKLFLVPGAAHCATNPLQPNGAFPNTNLAVMIDWVENAVEPSTLNATHLTGSNLGAQEQLCAWPLRPYYSGNATSPACVYDQ
KSIDSWMYDFNAYKLPLY
```

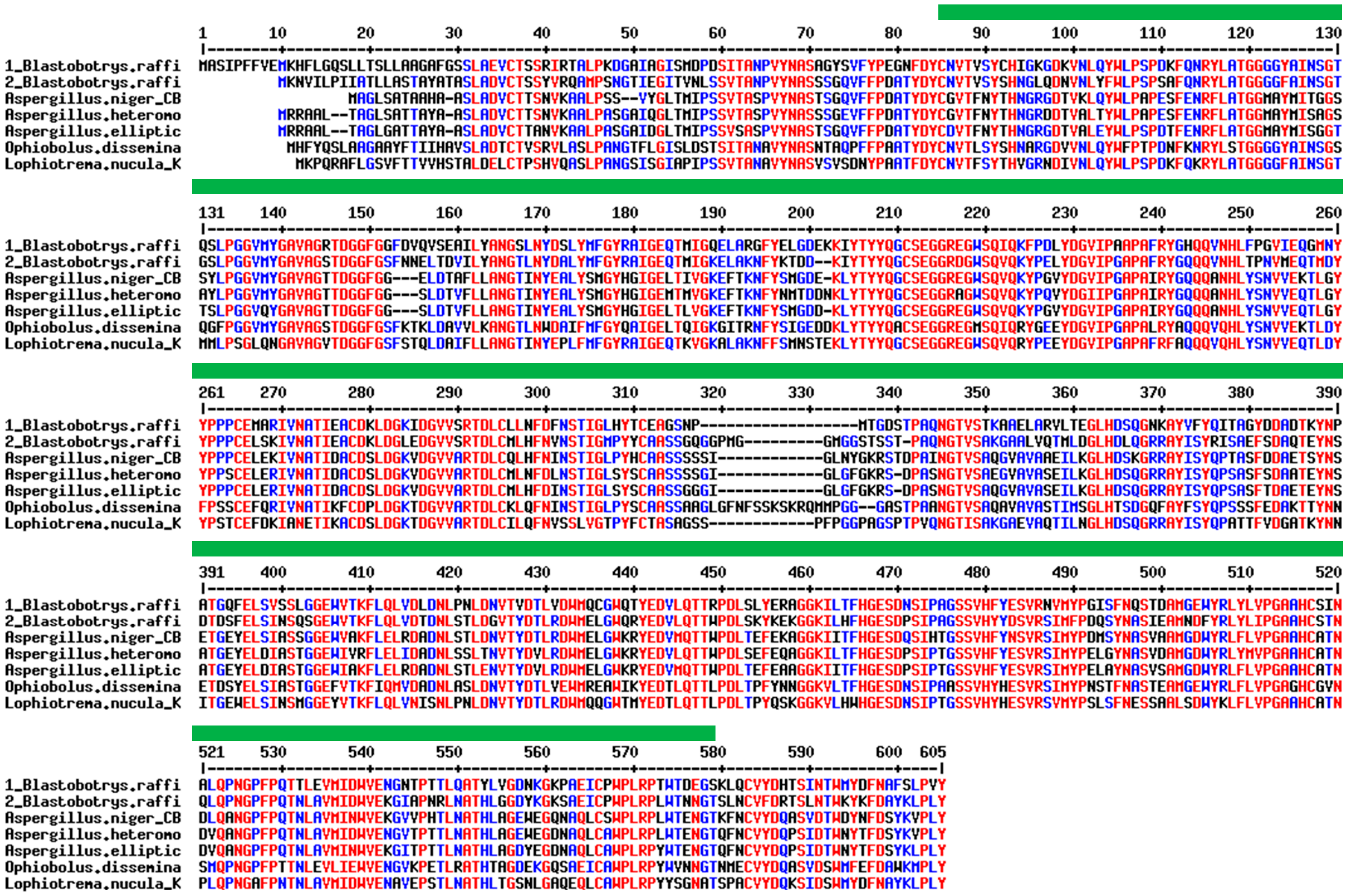

Alignments were performed with the Multalin server (http://multalin.toulouse.inra.fr/multalin/). The *B. raffinosifermentans* ARAD1A06094 and ARAD1A19822 predicted proteins were aligned with *Aspergillus niger* CBS 101883 XP_025455234.1, *Aspergillus heteromorphus* CBS 117.55 XP_025395144.1, *Aspergillus ellipticus* CBS 707.79 PYH90982.1, *Ophiobolus disseminans* KAF2833293.1 and *Lophiotrema nucula* KAF2119681.1 conserved proteins

Amino acids 85 to 561 of the *B. raffinosifermentans* Tan1 protein (ARAD1A06094) correspond to the pfam07519 conserved domain (de Vries et al.; 2002)
