# Supplementary material for: Development of a Vector Set for High or Inducible Gene Expression and Protein Secretion in the Yeast Genus Blastobotrys
Source: J Fungi (Basel). 2022 Apr 19;8(5):418. doi: 10.3390/jof8050418 (PMC9144253; doi:10.3390/jof8050418)
Supplement: Supplementary file 1 [file jof-08-00418-s001.zip › TableS1.pdf]

Table S1: Yeast strains used in this study

Yeast strains	Phenotype	Reference
Apiotrichum siamense L8in5 (CLIB 3781)	WT	This study
B. raffinosifermentans LS3	WT	Wartmann et al. ; 1995, Thomas et al. ; 2019
B. raffinosifermentans CBS 8335	WT	Thomas et al.; 2019
B. yvelinesensis nomen nudum L1-24 (CLIB 3782)	WT	This study
B. yvelinesensis nomen nudum L2-36 (CLIB 3783)	WT	This study
B. illinoisensis YB-1343	WT	Kurtzman et al. ; 2007
B. malaysiensis Y-6417	WT	Kurtzman et al. ; 2007
B. mokoenaii Y-27120	WT	Ravn et al.; 2021
CBS8335+rDNA::p*TEF1*-eYFP-*PHO5*t::rDNA	hp4d-*hph*	This study
CBS8335+rDNA::p*TDH3*-eYFP-*PHO5*t::rDNA	hp4d-*hph*	This study
CBS8335+rDNA::p*HSP26*-eYFP-*PHO5*t::rDNA	hp4d-*hph*	This study
CBS8335+rDNA::p*PCK1*-eYFP-*PHO5*t::rDNA	hp4d-*hph*	This study
CBS8335+rDNA::p*TPS1*-eYFP-*PHO5*t::rDNA	hp4d-*hph*	This study
CBS8335+rDNA::p*XYL1*-eYFP-*PHO5*t::rDNA	hp4d-*hph*	This study
CBS8335+*TDH3*::p*TDH3*-eYFP-*PHO5*t	hp4d-*hph*	This study
CBS8335+*TDH3*::p*TDH3-ABN1-PHO5*t	p*YlTEF1-nat*	This study
CBS8335+*AGU1*::p*TEF1-AGU1-PHO5*t	hp4d-*hph*	This study

CLIB numbers refer to the French CIRM-levures https://cirm-levures.bio-aware.com
