# Supplementary material for: Development of a Vector Set for High or Inducible Gene Expression and Protein Secretion in the Yeast Genus Blastobotrys
Source: J Fungi (Basel). 2022 Apr 19;8(5):418. doi: 10.3390/jof8050418 (PMC9144253; doi:10.3390/jof8050418)
Supplement: Supplementary file 1 [file jof-08-00418-s001.zip › TableS2.pdf]

Table S2: List of primers used in this study

**Number**	**Name**	**Sequence**
Promoter cloning		
1	YFP_ATG_BamHI	CC**GGATCC**CACAATGGTGAGCAAGGGCGAGG
2	YFP_STOP_NotI	CC**GCGGCCGC**TTACTTGTACAGCTCGTCCATG
3	HSP26_Prom_UpSalI	CAATCCTG**GTCGAC**GCGTAC
4	HSP26_Prom_DoBamHI	CC**GGATCC**TGTCGATACTGCTTTCAAGTAAC
5	PCK1_Prom_UpSalI	CC**GTCGAC**TCTACCTAGACCTAAGTATTAAAC
6	PCK1_Prom_DoBamHI	CC**GGATCC**TTTGAAAAGTTAATAAACAGATACT
7	TDH3_Prom_UpSalI	CC**GTCGAC**ATGATGAGATTGATTGATCAGAT
8	TDH3_Prom_DoBamHI	CC**GGATCC**TGTTAACAAGTTTTTATCAATTGC
9	TPS1_Prom_UpSalI	CC**GTCGAC**GGATACAAAGAACTACGTTTTAAG
10	TPS1_Prom_DoBamHI	CC**GGATCC**TGTTCGACTAATCGTCCGTTAAA
11	XYL1_Prom_UpXhoI	GG**CTCGAG**CTAGGTGATAAACATTGGTTATA
12	XYL1_Prom_DoBamHI	CC**GGATCC**TGCCTGAGGAAGATGCCAC
13	TDH3_Prom_UpExt_F	GAACATCAGTGACACTTGGCC
14	PHO5_Rev	GCCTGCAGATTTTAATCTTTCGGC
15	TEF1_For	TGGTCGACTTCAATCTATAATCAG
Secretion vectors construction and homologous and heterologous enzymes expression		
16	BrABN1_ATG_Bam	CC**GGATCC**ACAATGAAGAGCCAATTGATCG
17	BrABN1_SSV5_R	ACCCAACAATGGATTTGGAATTGGTTTACCTGCCACCACAGAGCTCATAGAG
18	BrABN1_V5_F	CCAAATCCATTGTTGGGTTTGGATTCA**ACTAGT**GCAAAGTCTAAGACCGATGATTC
19	BrABN1_STOP_NotI	GG**GCGGCCGC**TAAACGAGTTTTGGCCAACCGTC
20	BrAGU1_ATG_BamHI	CC**GGATCC**AAATGTGCATAGACTTCACAATGC
21	BrAGU1_SS6HIS_R	**CACGTG**ATGGTGATGGTGATGGCGCGCCGCCGCCAACCCTAAG
22	BrAGU1_6HIS_F	CGGCGCGCCATCACCATCACCAT**CACGTG**GAGAACGGCATAGATGGTTGG
23	BrAGU1_STOP_NotI	GG**GCGGCCGC**TAGTCTATGATTCCTCCAGGAAG
24	BrAGU1_DoExt_R	ATTGAGTCCAGAAAGGGGTC
25	BrAGU1_Verif_F	GGAGTATTCTGCAAGACTGG
26	AsAXH1_10F_SpeI_BssHII	CCACTAGT**GCGCGC**CCTCTCGTCTCCCACATTTAC
27	AsAXH1_972R_EagI_ FLAG_NotI	GG**GCGGCCGC**TATTTATCATCATCATCTTTATAATC**CGGCCG**CTTCTGCTGGATAATC
Phylogeny		
28	ITS1	TCCGTAGGTGAACCTGCGG
29	ITS4	TCCTCCGCTTATTGATATGC
30	NL1	GCATATCAATAAGCGGAGGAAAAG
31	NL4	GGTCCGTGTTTCAAGACGG
