# Supplementary material for: Development of a Vector Set for High or Inducible Gene Expression and Protein Secretion in the Yeast Genus Blastobotrys
Source: J Fungi (Basel). 2022 Apr 19;8(5):418. doi: 10.3390/jof8050418 (PMC9144253; doi:10.3390/jof8050418)
Supplement: Supplementary file 1 [file jof-08-00418-s001.zip › TableS3.pdf]

Table S3: List of the plasmids used and constructed in this study

**Plasmid Name**	**Base**	**Promoter**	**Expressed CDS**	**Yeast selection marker**	**Yeast integration site**
JMP1594	pDONR		e-YFP		
pARE12				hph	rDNA
pBS-SA-pTEF1-PHO5t		*TEF1*			
pTEF1-eYFP-PHO5t	pARE12	*TEF1*	e-YFP	hph	rDNA
pTDH3-eYFP-PHO5t	pARE12	*TDH3*	e-YFP	hph	rDNA
pHSP26-eYFP-PHO5t	pARE12	*HSP26*	e-YFP	hph	rDNA
pPCK1-eYFP-PHO5t	pARE12	*PCK1*	e-YFP	hph	rDNA
pTPS1-eYFP-PHO5t	pARE12	*TPS1*	e-YFP	hph	rDNA
pXYL1-eYFP-PHO5t	pARE12	*XYL1*	e-YFP	hph	rDNA
pTDH3-eYFP-PHO5t	pBS-SA	*TDH3*	e-YFP	hph	*TDH3*
pTDH3-V5-ABN1-PHO5t	pBS-SA	*TDH3*	*BrABN1* (ARAD1D18216g)	nat	*TDH3*
pTEF1-6xHis-AGU1-PHO5t	pBS-SA	*TEF1*	*BrAGU1* (ARAD1D23848g)	hph	*AGU1*
pTDH3-SS-V5-AXH1-Flag-PHO5t	pBS-SA	*TDH3*	*AsAXH1*	nat	*TDH3*
pTEF1-SS-AXH1-Flag-PHO5t	pBS-SA	*TEF1*	*AsAXH1*	nat	rDNA
