# Supplementary material for: Development of a Vector Set for High or Inducible Gene Expression and Protein Secretion in the Yeast Genus Blastobotrys
Source: J Fungi (Basel). 2022 Apr 19;8(5):418. doi: 10.3390/jof8050418 (PMC9144253; doi:10.3390/jof8050418)
Supplement: Supplementary file 1 [file jof-08-00418-s001.zip › TableS4.pdf]

Table S4: Sequence identity in the ITS and D1D2 domains of the 26S rRNA gene of *Blastobotrys* species

		**Y6417**	**Y27120**	**YB1343**	**L1-24**	**L2-36**
B. malaysiensis	**Y6417**	1134	99%	97%	97%	97%
			0%	0%	0%	0%
B. mokoenaii	**Y27120**	1125	1136	97%	97%	97%
		2		0%	0%	0%
B. illinoisensis	**YB1343**	1114	1113	1132	97%	97%
		8	6		0%	0%
B. yvelinesensis	**L1-24**	1113	1113	1111	1134	100%
		10	8	4		0%
B. yvelinesensis	**L2-36**	1113	1113	1111	1134	1134
		10	8	4	0	

Blue coloured cells represent the number and percentage of nucleotide identity
Yellow coloured cells indicated the number of gaps and associated percentage.
