# Supplementary figures and images for: Development of a Vector Set for High or Inducible Gene Expression and Protein Secretion in the Yeast Genus Blastobotrys
Source: J Fungi (Basel). 2022 Apr 19;8(5):418. doi: 10.3390/jof8050418 (PMC9144253; doi:10.3390/jof8050418)

**Figure S4: EZ-blue gel corresponding to Figure 2A**

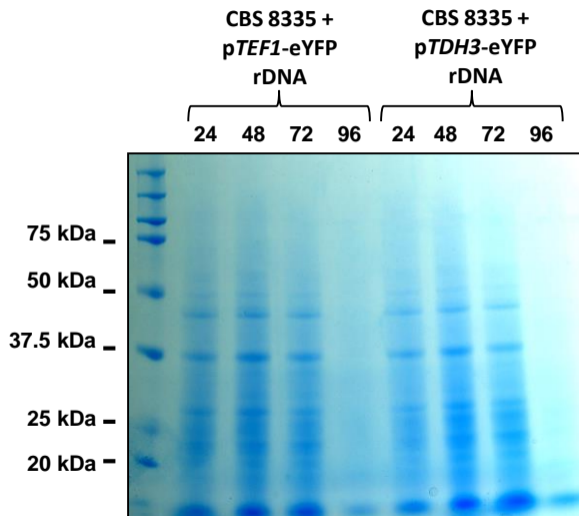

Supplement: Supplementary file 1 [file jof-08-00418-s001.zip › FigureS4.pdf]
